# Autophagy is essential for survival and function of polyploid giant cancer cells under therapeutic stress

**DOI:** 10.1016/j.canlet.2026.218431

**Published:** 2026-03-14

**Authors:** D. Ghosh, B. White, X. Lu, M. Perricone, M. Zhou, B. Xuan, Y. Li, Y. Wang, M.R. Dawson

**Affiliations:** a Department of Molecular Biology, Cell Biology, and Biochemistry, Brown University, Providence, RI, 02912, USA; b School of Engineering, Brown University, Providence, RI, 02912, USA; c Institute for Biology, Engineering and Medicine, Brown University, Providence, RI, 02912, USA; d Department of Pathology and Laboratory Medicine, Brown University, Providence, RI, 02912, USA; e Brown University, Providence, RI, 02912, USA

**Keywords:** PGCC, Paclitaxel, Autophagy, Micronuclei, Vimentin, p62, Daughter cells

## Abstract

Polyploid giant cancer cells (PGCCs) are enlarged, multinucleated tumor cells that arise in response to stressors such as chemotherapy and are increasingly recognized as key drivers of recurrence and metastasis in aggressive cancers. Found in triple-negative breast cancer (TNBC) and ovarian cancer (OC), PGCCs can survive cytotoxic therapy in a dormant state and later produce chemoresistant progeny through amitotic budding. Here, we investigated the role of autophagy in paclitaxel (PTX)-induced PGCC survival, nuclear maintenance, and migration. PGCCs generated from MDA-MB-231 and HEY cells were significantly larger, more heterogeneous, and more resistant to PTX than parent cells. Transcriptomic profiling revealed enrichment of metabolic and cytoskeletal pathways, with strong upregulation of autophagy-related genes, including *SQSTM1 (P62)*, *LC3*, and *LAMP1*. PGCCs exhibited elevated oxidative stress and marked induction of mitochondrial superoxide dismutase 2 (*SOD2*). p62 was localized near micronuclei, and prolonged autophagy inhibition with Bafilomycin A1 reduced nuclear size, heterogeneity, and micronuclei number. PGCCs also displayed a dispersed vimentin intermediate filament network that scaffolded autophagic structures; autophagy inhibition impaired migration in PGCC-derived daughter cells. These findings identify autophagy as a critical process sustaining PGCC survival, structural integrity, and motility, and suggest that targeting autophagy may disrupt PGCC-driven recurrence in aggressive cancers.

## 1. Introduction

A major challenge in treating malignant cancer is its inherent heterogeneity, which generates a complex molecular landscape containing subpopulations of highly invasive and chemoresistant cells [1–3]. Among these, polyploid giant cancer cells (PGCCs) are a drug-resistant, often dormant subpopulation frequently found in late-stage aggressive cancers, including triple-negative breast cancer (TNBC) and ovarian cancer (OC) [4–6]. Despite advances in targeted therapies such as PARP inhibitors, antiangiogenic agents, and immune checkpoint inhibitors, chemotherapy remains the standard of care for both TNBC and OC [7,8]. PGCCs can survive treatment and later re-enter the cell cycle, producing chemoresistant progeny through amitotic budding [9–11]. Because of their role in recurrence and therapy resistance, PGCCs represent a critical target for therapeutic intervention [12,13].

Chemotherapeutic agents that target rapidly dividing cancer cells—particularly microtubule-targeting drugs like paclitaxel (PTX)—select for non-mitotic PGCCs formed through mitotic slippage, endoreplication, or cell fusion [14,15]. These multinucleated cells must adapt to the structural and metabolic demands of their large, abnormal morphology as well as oxidative stress and DNA damage induced by treatment [16]. Persistent stress activates survival-promoting stress response pathways, including autophagy, a conserved mechanism for degrading and recycling damaged cellular components to maintain homeostasis [17,18]. PGCCs have been shown to induce autophagy under hypoxic conditions, using processes such as mitophagy and lipophagy to mitigate oxidative stress and repair mitochondrial damage [19]. By breaking down damaged components, PGCCs can generate precursors for oxidative phosphorylation (OXPHOS), supporting the higher ATP demands of their enlarged structure and motility [18]. This metabolic adaptation is often accompanied by increased expression of antioxidant enzymes such as *SOD2*, which reduces mitochondrial reactive oxygen species (ROS) and further promotes survival [20,21].

Structurally, PGCCs display unique cytoskeletal adaptations [22,23]. In addition to altered actin architecture and elevated stiffness, PGCCs possess a dispersed network of vimentin intermediate filaments (VIFs) that provide structural support for their enlarged morphology and facilitate persistent migration [24,25]. VIFs act as scaffolds for lysosomes and autophagosomes, enabling their fusion during autophagy and enhancing autophagic flux [26,27]. Zinc binding to a specific cysteine residue (C328) in vimentin stabilizes VIF networks, while zinc deficiency disrupts these filaments, impairs lysosomal integrity, and promotes aggresome formation [28,29]. The vimentin–autophagy axis may therefore be central to PGCC survival by integrating structural stability, organelle positioning, antioxidant defense, and energy production needed for polarized migration.

In this study, we investigated the role of autophagic flux in PGCC survival and migratory persistence, and how altered vimentin organization regulates the spatial distribution of autophagosomes. Using TNBC (MDA-MB-231) and OC (HEY) cell lines, we found that PTX-induced PGCCs show elevated expression of autophagy-related genes and generate chemoresistant daughter cells. Autophagy was upregulated in both PGCCs and their progeny and contributed to PGCC motility. Pharmacologic inhibition of autophagy reduced PGCC size and diminished migratory persistence, supporting the therapeutic potential of targeting vimentin-regulated autophagy to limit PGCC formation, survival, and dissemination.

## 2. Materials and methods

### 2.1. Cell culture

Human TNBC cell line MDA-MB-231 and OC cell line HEY were purchased from ATCC. MDA-MB-231 cells were maintained in DMEM/Ham’s F-12 50/50 Mix (Corning) supplemented with 10% fetal bovine serum (FBS; Bio-Techne) and 1% penicillin–streptomycin (Corning). HEY cells were cultured in RPMI-1640 medium (Corning) supplemented with 10% FBS and 1% penicillin–streptomycin. All cultures were maintained in a humidified incubator at 37 °C with 5% CO_2_.

To enrich for the polyploid giant cancer cell (PGCC) subpopulation, cells were grown to 80–90% confluency and treated with 500 nM PTX for 16h (MDA-MB-231) or 72h (HEY). Cells were then returned to standard culture medium for an additional 7 days before downstream experiments.

### 2.2. Daughter cell isolation

PTX induced PGCCs from MDA-MB-231 and HEY cells were moved to a 24 well plate and maintained in normal media for an extended period of time (~6–12 weeks). Cells were checked weekly to identify appearance of colonies of daughter cells. Cells were then moved to a larger culture dish and used for downstream experiments.

### 2.3. Chemical preparations

PTX, carboplatin (CPTN), and Bafilomycin A1 (BafA1) were purchased from Cayman Chemical. PTX and BafA1 stocks were prepared in dimethyl sulfoxide (DMSO), while CPTN stocks were prepared in water. All stocks were aliquoted and stored at − 20 °C until use.

### 2.4. Tissue array analysis

H&E-stained TNBC (BR1202a) and OC (OVCA1004a) tissue microarrays were obtained from US Biomax Inc. The TNBC array included only four biopsy samples for stage I and did not contain any samples from advanced stage (stage IV) cases. The majority of samples in the OVCA tissue microarray were from patients diagnosed with high-grade serous ovarian carcinoma (HGSOC). In the OVCA array, only two samples came from stage IV cases; therefore, these were combined with stage 3 samples for analysis. Additional H&E-stained benign breast and ovarian tissue sections were provided by Dr. Yihong Wang. Slides were imaged at 40 × magnification (dry, without oil) using an Olympus VS200 slide scanner at the Leduc Bioimaging Facility. Regions of interest (cancerous and control) were manually traced and processed using the CellPose-SAM segmentation model in Python [30]. Nuclei touching image borders were excluded to avoid edge effects in nuclear area calculations. PGCCs were defined as nuclei ≥3 × the median nuclear area of the cancerous population, with cutoffs of 797 μm^2^ for OVCA (HGSOC) and 814 μm^2^ for TNBC [4].The analysis of the percentage of positive Ki67 cells in TNBC array was obtained from US Biomax Inc. It includes data for stage 1 (n = 2), stage 2 (n = 43), and stage 3 (n = 6) cancers. The detailed list of sample information and number of areas analyzed are included in Supplementary Table 1.

### 2.5. MTT assay

Cells were seeded in 96-well tissue culture plates (Corning) and incubated overnight. Cells were treated with PTX or CPTN for 72 h; DMSO and water served as vehicle controls. After treatment, media was replaced with 120 μL of MTT reagent (1 mg/mL in culture media) and incubated for 4 h at 37 °C. MTT solution was then removed, and 150 μL MTT solvent (4 mM HCl in isopropanol) was added. Plates were shaken for 15 min before absorbance measurement at 595 nm.

### 2.6. Microarray analysis

Parent and PTX-induced PGCC MDA-MB-231 cells (N = 4 biological replicates) were washed in PBS, lysed in RiboZOL, and stored at −80 °C. RNA was extracted via standard chloroform–isopropanol precipitation and amplified from 100 ng total RNA using the Affymetrix Sensation Plus FFPE amplification kit. Labeled cDNA was hybridized to Affymetrix Clariom-D microarrays and scanned at the Brown University Genomics Core Facility per manufacturer’s protocols.

Data were normalized in Transcriptome Analysis Console (Affymetrix), followed by principal component analysis. Genes with >2-fold differential expression (DEGs), p < 0.05, and false discovery rate (FDR) < 0.1 between PGCCs and parent cells were identified. Significantly enriched canonical pathways were analyzed using Ingenuity Pathway Analysis (IPA) to calculate activation z-scores. A list of DEGs and IPA pathway analysis results are provided in Supplementary Table 2.

### 2.7. RNA isolation, cDNA synthesis, and qRT-PCR

RNA isolation, cDNA synthesis, and qRT-PCR reagents were from Bio-Rad. Cells were lysed in TRIzol, and RNA was extracted by phenol–chloroform separation, precipitated with isopropanol, washed in 70% ethanol, and resuspended in nuclease-free water. One microgram of RNA was reverse transcribed using the iScript gDNA Clear cDNA synthesis kit (Bio-Rad).

qRT-PCR was performed with SYBR Green Supermix (Bio-Rad) on a CFX instrument. *β-ACTIN* was used for normalization, and fold changes were calculated using the (-ΔΔCt) method. Primer sequences:

***β-ACTIN*:** F 5′-AGAGCTACGAGCTGCCTGAC-3′; R 5′-AGCACTGTGTTGGCGTACAG-3′

***P62/SQSTM1*:** F 5′-AGGCGCACTACCGCGAT-3′; R 5′-GTCACTGGAAAAGGCAACC-3′

***LC3B*:** F 5′-GAGCAGCATCCAACCAAA-3′; R 5′-GTCTCCTGGAGGCATA-3′

***LAMP1*:** F 5′-TCTCAGTGAACTACGACACCA-3′; R 5′-AGTGTATGTCCTCTTCCAAAAGC-3′

***SOD2:*** F 5′- GCTCCGGTTTTGGGGTATCTG-3’; R 5′-GCGTTGATGTGAGGTTCCAG-3’

### 2.8. Immunofluorescence staining

Cells were seeded on glass coverslips in 24-well plates and cultured for 24 h. Samples were fixed in 4% paraformaldehyde (Sigma-Aldrich), washed with PBS, and permeabilized in microscopy buffer (5 g BSA, 0.5 g saponin, 0.5 mL 20% w/v sodium azide in 500 mL PBS; filtered through a 0.22 μm filter). Samples were incubated overnight with primary antibodies: vimentin (1:200, Cell Signaling), p62/SQSTM1 (1:100, Invitrogen), and SOD2 (1:100, Invitrogen). Secondary antibodies were applied along with Hoechst 33342 and rhodamine-conjugated phalloidin (1:400, Invitrogen) for 1 h at room temperature. Coverslips were mounted with Fluoromount-G and imaged using a Nikon Eclipse Ti inverted fluorescence microscope or Olympus Yokogawa spinning disk confocal microscope.

### 2.9. Immunofluorescence image analysis

Images taken with Nikon Eclipse Ti microscope at 10x magnification were analyzed in CellProfiler (Broad Institute). Cell boundaries were manually traced to quantify marker intensity and distribution. For radial distribution, cells were divided into eight concentric bins and mean fractional intensity per bin was calculated. PGCCs were identified as nuclei ≥2.5 × the average nuclear size of the parent population (Hoechst-stained) [24,25]. Colocalization coefficient between p62 and DAPI was measured in CellProfiler.

### 2.10. Live-cell imaging with CellROX

Cells were seeded in 35 mm dishes (10–15 × 10^3^ cells/dish) and stained 24 h later with Hoechst 33342, CellROX Red, and MitoTracker Deep Red. Imaging was performed at 30 × magnification. Cells were manually traced in FIJI, and single-cell intensity analysis was conducted in CellProfiler.

### 2.11. BafA1 treatment of cells

For BafA1 treatment of cells, concentration was selected based on preliminary dose-response experiments comparing lower (~10 nM) and higher (~100 nM) concentrations. For short-term assays such as the wound healing assay, we used 100 nM to ensure effective autophagy inhibition during the limited experimental window. For longer or chronic treatments, we used the lower dose to minimize potential toxicity. Both concentrations fall within the commonly reported in vitro range (10–200 nM) for autophagy inhibition [31].

For chronic inhibition of autophagy, cells were seeded at a density of 25,000 cells per well in a 24 well plate. Next day, cells were treated with 500 nM PTX for 18 h before adding vehicle control (DMSO) and BafA1 (10 nM) for 72 h. DMSO/BafA1 treatment media was renewed for another 72 h before switching to normal media. Cells were then analyzed after 48 h.

### 2.12. Live-cell imaging with calcein AM

Cells were incubated with Hoechst 33342 (1:10,000 dilution of 10 mM stock), Calcein AM (1:2000 of 1 mM stock) and propidium iodide (1:500 of 1 mg/ml stock) for 30 min in the cell culture incubator before imaging on the Nikon Eclipse Ti inverted fluorescence microscope maintained at 37 °C with 5% CO_2_.

### 2.13. Crystal violet staining

Cells were fixed in 10% formalin for 10 min at room temperature before washing and staining with 0.5% (w/v) crystal violet. After washing, stained cells were imaged at 4x using Olympus Color Microscope. We measured crystal violet absorbance at 590 nm wavelength using Varioskan Lux plate reader.

### 2.14. Immunofluorescent TUNEL assay

To determine apoptosis in PGCCs, we used Click-iT^™^ Plus TUNEL Assay Kit (Invitrogen) as per manufacturer’s recommendation. Briefly, HEY parent cells and PGCCs cultured on 12 mm coverslips were treated with vehicle control or 100 nM BafA1 for 24 h, then fixed with 4% paraformaldehyde and permeabilized using 0.25% Triton-X in PBS. For positive control, fixed and permeabilized PGCC samples were treated with DNASE I for 30 min at room temperature. The TdT reaction was performed for 60 min, then a 30-min Click-it reaction with Alexa Fluor 594 at 37 °C. Vimentin and P62 were detected using the immunofluorescence staining protocol described above. Cells were imaged at 30x with Olympus Yokogawa spinning disk confocal microscope.

### 2.15. Scratch wound assay

Cells were grown to confluence in 24-well plates pre-coated with 20 μg/mL type I rat tail collagen (Corning). Wounds were made using a 10 μL pipette tip in a cross pattern, washed three times with PBS, and incubated in Hoechst-containing medium (1 μg/mL) for 30 min. Images were captured at 0 h and 12 h using a Nikon Eclipse Ti or Olympus Yokogawa spinning disk confocal microscope in a 37 °C, 5% CO_2_ environmental chamber. PGCCs were identified and quantified by nuclear area using CellProfiler.

### 2.16. Statistics

Statistical analysis was performed in GraphPad Prism 10. Data represent pooled measurements from ≥300 cells per condition from independent biological replicates (N). Results are expressed as mean ± S.D. or mean ± S.E.M. Comparisons between two groups were made using two-tailed unpaired Student’s t-tests with Welch’s correction (unequal variance). Statistical analysis for the tissue arrays was conducted using the Mann-Whitney *U* test to compare group medians. For more than two groups, one-way ANOVA with Tukey’s multiple comparisons test was used. Significance was reported as p ≤ 0.05 (*), p ≤ 0.01 (**), p ≤ 0.001 (***), or p ≤ 0.0001 (****).

## 3. Results

### 3.1. Paclitaxel-induced PGCCs are distinctively larger and resistant compared to parent cells

**Fig. 1.**
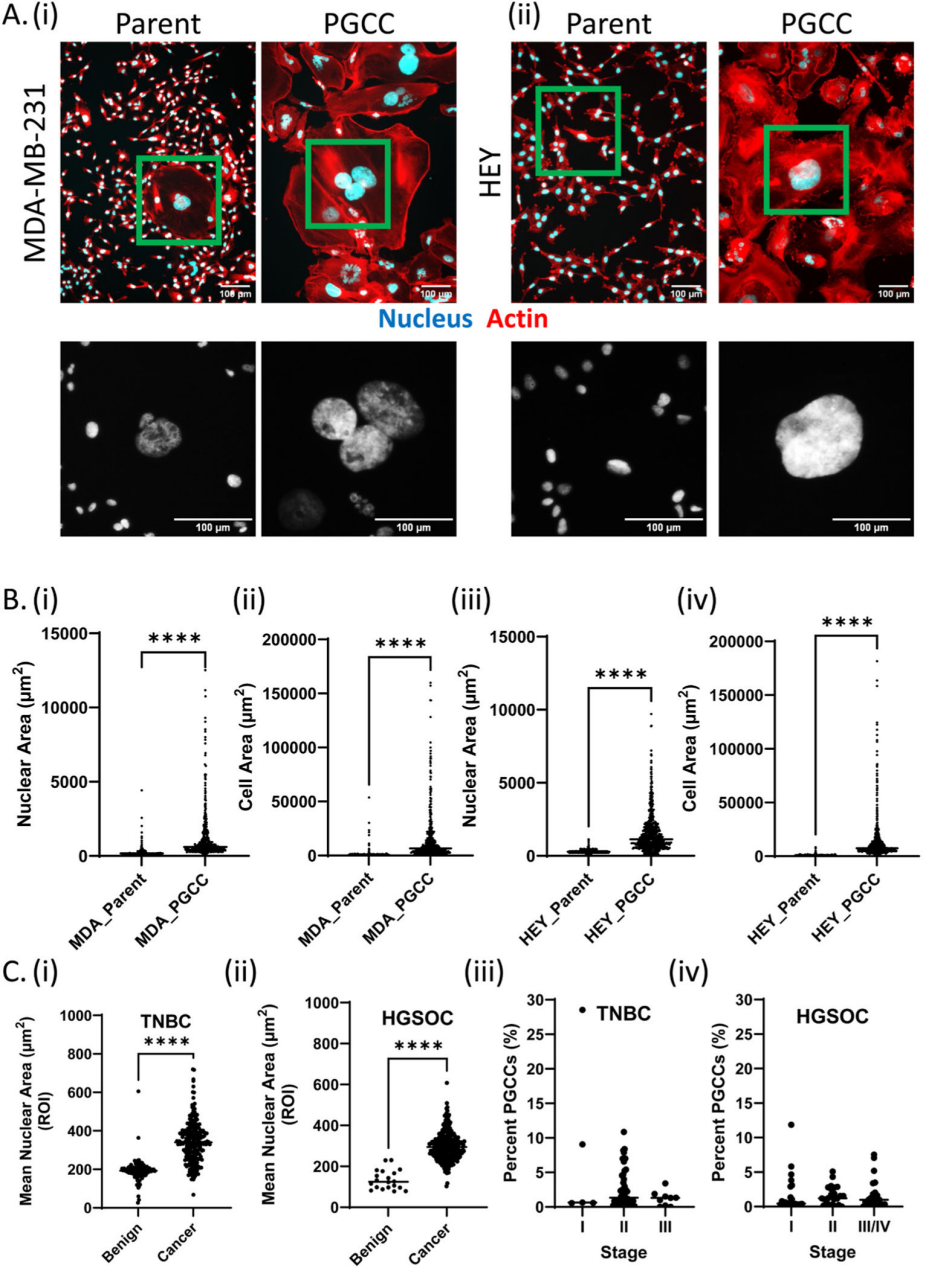
Therapy induced PGCCs displayed increased size. (A-B) MDA-MB-231 and HEY cells were exposed to 500 nM Paclitaxel (PTX) for 18 and 72 h, respectively, followed by a 7-day recovery period. (A) Images of cells fixed and stained with DAPI (blue) and phalloidin (red) show that PTX-induced PGCCs (i-ii, right) exhibited increased nuclear and cell size compared to the parent cells (i-ii, left) (Scale bars = 100 μm). (B) Quantification of nuclear (i, iii) and cell (ii, iv) areas for parent cells and PGCCs from immunofluorescence images indicated increases in both measurements, with nuclear area increasing more than 5-fold and cell area increasing more than 10-fold. Graphical and statistical analysis were computed in GraphPad Prism for n > 500 cells (N = 2). An unpaired *t*-test with Welch’s correction was performed to calculate statistical significance indicated as (***) for p < 0.0001. (C) We identified and quantified PGCCs in triple negative breast cancer (TNBC) and high grade serous ovarian cancer (HGSOC) using H&E-stained tissue microarrays. Nuclear areas of tumor regions in TNBC and HGSOC tissues were significantly higher compared to benign tissues (i-ii). We also identified percent PGCCs per biopsy sample in all stages of the disease (iii-iv). Graphical and statistical analysis were computed in GraphPad Prism for regions of interest (ROI) comprising greater than 10,000 cells per group for tissue microarray data analysis. Statistical analysis for the tissue arrays was performed using nonparametric unpaired Mann-Whitney U tests with significance indicated as (****) for p < 0.0001. (For interpretation of the references to colour in this figure legend, the reader is referred to the Web version of this article.)

To generate a stable, enriched population of PGCCs in MDA-MB-231 (TNBC) and HEY (OC) cells, we treated cells with 500 nM PTX followed by a 7-day recovery period. PTX-induced PGCCs displayed a marked increase in both nuclear and cell size compared to the respective parent cell lines (Fig. 1A and B, Supplementary Fig. 1). Quantification of immunofluorescence images in CellProfiler (n > 500 cells, N = 2) showed more than a 5-fold increase in nuclear area and a 10-fold increase in cell area in both cancer types (Fig. 1B). Heterogeneity in nuclear size, measured by coefficient of variation (CV), was significantly higher in PGCCs for both cell lines (Supplementary Fig. 1A). For cell area, CV was higher in HEY PGCCs compared to parent cells, whereas parent MDA-MB-231 cells showed a higher CV than their PGCC counterparts— likely due to the presence of naive PGCCs within the untreated population.

To assess DNA content, we measured the integrated intensity of nuclear staining as a proxy for total DNA (Supplementary Fig. 1B). Frequency distribution analysis revealed a clear shift toward higher nuclear content in PGCCs compared to parent cells (Supplementary Fig. 1C). Despite these differences in size, correlation analysis showed a significant (p < 0.0001) positive relationship between nuclear and cell areas in both parent and PGCC populations (Supplementary Fig. 1D).

Chemoresistance of the stable PGCCs was tested by re-treating cells with PTX or CPTN and quantifying viability via MTT assay (Supplementary Fig. 1E). PGCCs remained highly resistant to PTX, showing minimal cell death after 72 h. While PGCCs were more sensitive to CPTN than to PTX, their IC_50_ values remained higher than those of the parent cells.

We next examined the prevalence of PGCCs in TNBC and OC patient tissues. H&E-stained tumor and benign sections were analyzed using CellProfiler to quantify nuclear area (Fig. 1C, Supplementary Fig. 2). The average nuclear area in cancerous tissues was significantly larger than in matched benign tissues. PGCCs were defined as nuclei ≥3 × the median nuclear area of the cancer cell population and comprised approximately 1–2% of tumor cells overall, comparable to the naïve PGCC frequency observed in untreated cell lines. Most tumors exhibited a PGCC frequency of ~1–2%. However, 20% of TNBC and 15% of OC samples demonstrated elevated PGCC levels (2–5%). A subset of tumors showed particularly high PGCC abundance (>5%), observed in 20% of TNBC and 6% of OC samples. In TNBC, larger PGCCs were observed in stage I–II tumors compared to stage III tumors. In OC (HGSOC), both mean PGCC nuclear area and PGCC frequency per ROI increased modestly with tumor stage, although these differences did not reach statistical significance.

### 3.2. Autophagy is significantly upregulated in PGCCs

**Fig. 2.**
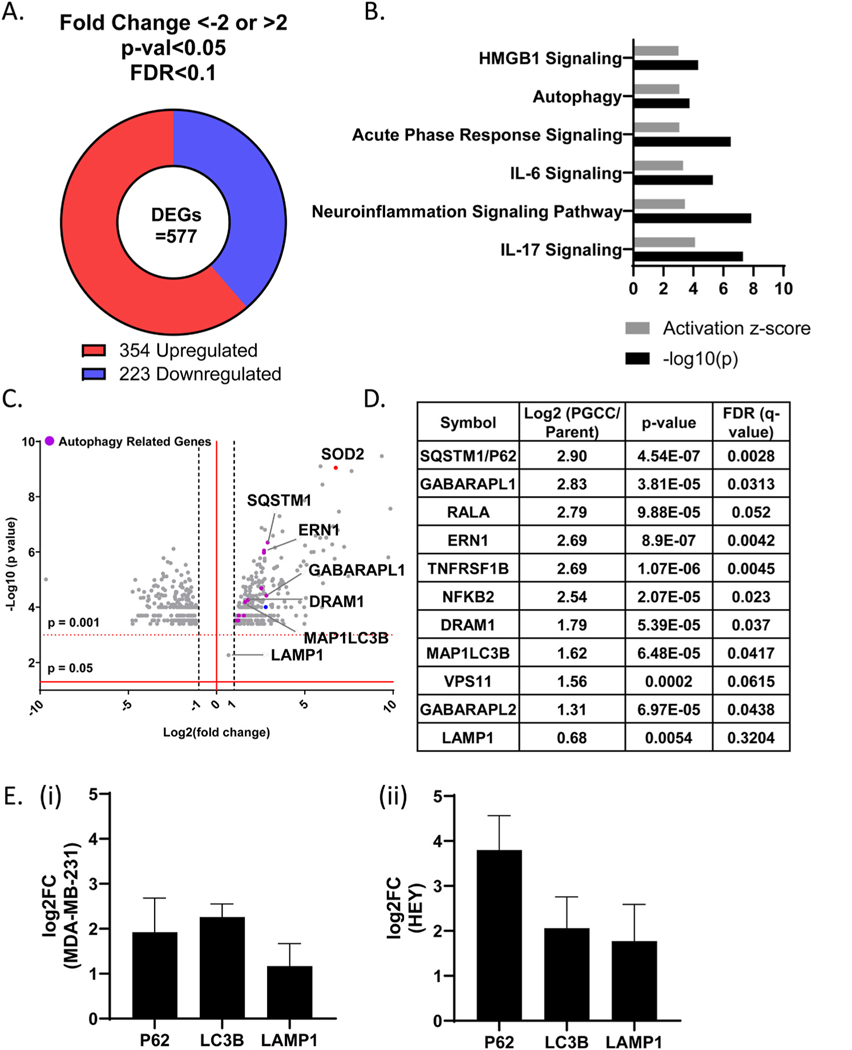
Autophagy upregulated in PTX-treatment induced PGCCs. (A) Affymetrix Clariom-D microarrays were used to measure genome wide transcriptional changes in paclitaxel (PTX) induced PGCCs compared to parent MDA-MB-231 cells. The number of significantly regulated genes (|fold change| > 2, p < 0.05, FDR <0.1) between parent and PGCCs was determined using the Transcriptome Analysis Console (TAC). Red and blue indicate the number of genes up- or downregulated in P GCCs compared to parent MDA-MB-231 cells (N = 4). (B) Ingenuity pathway analysis (IPA) of significantly upregulated (z-score>2) canonical pathway showed autophagy is increased in PGCCs compared to parent cells. (C-D) Autophagy-related genes are indicated in the volcano plot illustrating significantly upregulated genes (C), while their corresponding fold changes, p-values, and false discovery rates (FDR) are presented in panel D. (E) Quantitative real-time PCR (qRT-PCR) analysis confirmed elevated expression of autophagy and lysosomal genes in PGCCs relative to parent MDA-MB-231 (N = 4) and HEY (N = 3) cell lines. Bar graphs display mean ± S.E.M. (For interpretation of the references to colour in this figure legend, the reader is referred to the Web version of this article.)

We next compared gene expression profiles between MDA-MB-231 parent cells and PTX-induced PGCCs using Affymetrix Clariom-D microarrays (N = 4). Principal component analysis and unsupervised hierarchical clustering in the Affymetrix Transcriptome Analysis Console (TAC) revealed a distinct transcriptional signature for PGCCs (Supplementary Fig. 2A–B). Applying significance thresholds of p <0.05 and false discovery rate (FDR) < 0.1 identified 354 upregulated and 223 downregulated genes in PGCCs relative to parent cells (Fig. 2A). As expected, many of the downregulated genes were associated with cell cycle regulation (Supplementary Fig. 2C).

Gene ontology analysis for cellular components indicated enrichment of genes involved in metabolic processes and cytoskeletal organization in PGCCs (Supplementary Fig. 4A). Ingenuity Pathway Analysis (IPA; Qiagen) of significantly regulated pathways (activation z-score ≥2 or ≤ − 2) identified autophagy as one of the most strongly upregulated pathways in PGCCs (z-score = 3.05) (Fig. 2B; Supplementary Fig. 4B). Thirteen autophagy-related genes showed increased expression, including *SQSTM1 (P62)* and *DRAM1* (Fig. 2C and D).

To validate the microarray results, we performed qRT-PCR in both MDA-MB-231 and HEY cells for P62, LC3B and LAMP1. In the microarray analysis, LAMP1 expression showed a 1.6-fold increase (log2FC = 0.68, p < 0.05), but did not meet the initial false discovery rate (FDR) cutoff of 0.1 (FDR = 0.32) used for primary differential expression analysis. Despite not passing this stringent threshold, LAMP1 was consistently elevated in PGCCs and was therefore included for targeted validation. Consistent with the transcriptomic data, autophagy markers *P62* and *LC3B* were significantly upregulated in PGCCs compared to parent cells in both lines. The late lysosomal marker *LAMP1* also increased in PGCCs (Fig. 2E).

### 3.3. PGCCs are under severe oxidative stress

**Fig. 3.**
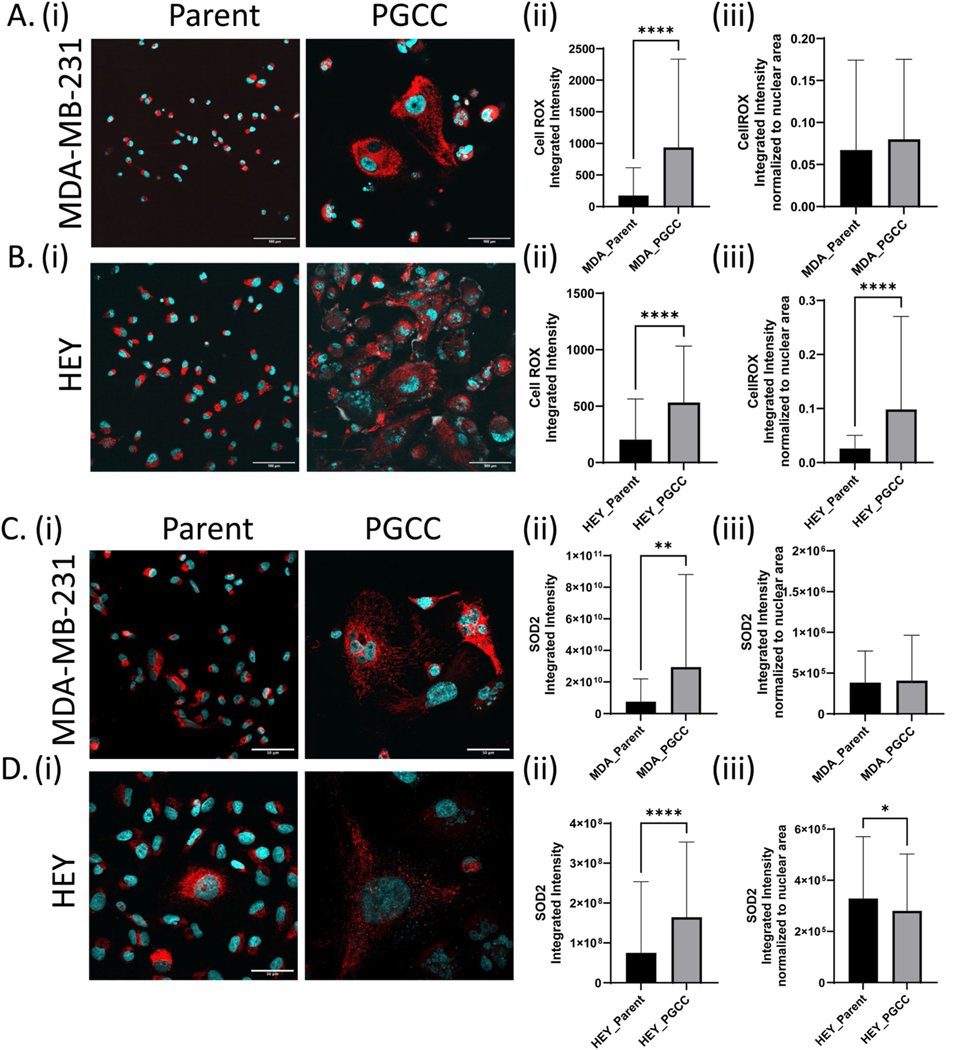
Increased oxidative stress in paclitaxel induced PGCCs compared to parent cells. (A-B) Representative live-cell images of MDA-MB-231 (A-i) and HEY (B-i) parent cells and PGCCs stained with CellROX dye to probe for reactive oxygen species (ROS) (Scale bars = 100 μm). To assess total oxidative stress per cell, integrated CellROX fluorescent intensity was quantified for individual cells (n > 50 cells per group; N = 3). PGCCs exhibited a significant increase in total integrated intensity compared to parent cells. (A-ii, B-ii). When normalized to nuclear area, CellROX levels were comparable between MDA-MB-231 PGCCs and parent cells but remained significantly elevated in HEY PGCCs relative to parent cells. (A-iii, B-iii). (C-D) Because oxidative stress is often associated with increased expression of antioxidant enzymes such as SOD2, we next quantified per-cell integrated SOD2 intensity using immunofluorescence analysis of antibody-stained cells (n > 50 cells per group; N = 2). Integrated SOD2 intensity was higher in PGCCs for both cell types (C-ii, D-ii), however, normalization to nuclear area revealed comparable levels in MDA-MB-231 cells and lower levels in HEY PGCCs compared to parent cells (C-iii, D-iii). Bar graphs display mean ± S.D. Statistical analysis used an unpaired *t*-test with Welch’s correction, with significance shown as (*) for p < 0.05., (**) for p < 0.01 and (****) for p < 0.0001.

Increased autophagy can serve as a cytoprotective function in response to chemotherapeutic stress, particularly under conditions that generate excessive reactive oxygen species (ROS) through mitochondrial dysfunction, impaired antioxidant defenses, or disrupted DNA replication. Cancer cells—and especially PGCCs—often upregulate antioxidant defenses to buffer ROS, although this typically results in a higher but tolerable oxidative state rather than complete ROS elimination. To determine whether ROS levels remain elevated in PTX-induced PGCCs and how oxidative stress may be linked to the PGCC phenotype and autophagy activation, we assessed intracellular ROS in PTX-induced PGCCs using live-cell microscopy. MDA-MB-231 and HEY cells were stained with CellROX, a live-cell indicator of ROS and imaged to assess oxidative stress at the single-cell level (Fig. 3A and B). In parent cells, CellROX fluorescence was primarily localized to the perinuclear region, consistent with mitochondrial localization. In PGCCs, staining was more widely distributed throughout the cytoplasm but remained enriched near mitochondria. Quantification of total ROS levels, based on integrated CellROX intensity per cell in CellProfiler, revealed significantly higher ROS levels in PGCCs compared to parent cells in both cancer types (Fig. 3A and B). When adjusted for the larger nuclear area of PGCCs, CellROX intensity was higher in HEY cells, while in MDA-MB-231 cells it remained comparable to that of their respective parent cells.

Given the association between oxidative stress and induction of antioxidant defenses, we next examined SOD2 expression. Microarray analysis demonstrated strong upregulation (>100-fold) of SOD2, which encodes mitochondrial superoxide dismutase 2, a key regulator of mitochondrial ROS detoxification (Fig. 2C). Increased SOD2 expression in PGCCs was confirmed by qRT-PCR in both cell lines (Supplementary Fig. 4C). Immunofluorescence staining revealed SOD2 localization patterns similar to those observed in the live-cell ROS assay, with perinuclear enrichment and broader cytoplasmic distribution in PGCCs. Quantification of integrated SOD2 intensity confirmed significantly higher overall expression in PGCCs compared to parent cells (Fig. 3C and D). However, when intensity was normalized to nuclear area, SOD2 levels were lower in HEY PGCCs compared to parent cells, and comparable to parent cells in MDA-MB-231 cells. Integrated and area-normalized assessments of CellROX and SOD2 provide important insights regarding PGCCs subjected to stress. This is particularly relevant for large, polyploid cells, where both the overall quantity within the cell and its spatial distribution may have significant biological implications.

### 3.4. Distribution of vimentin and p62 in PGCCs

**Fig. 4.**
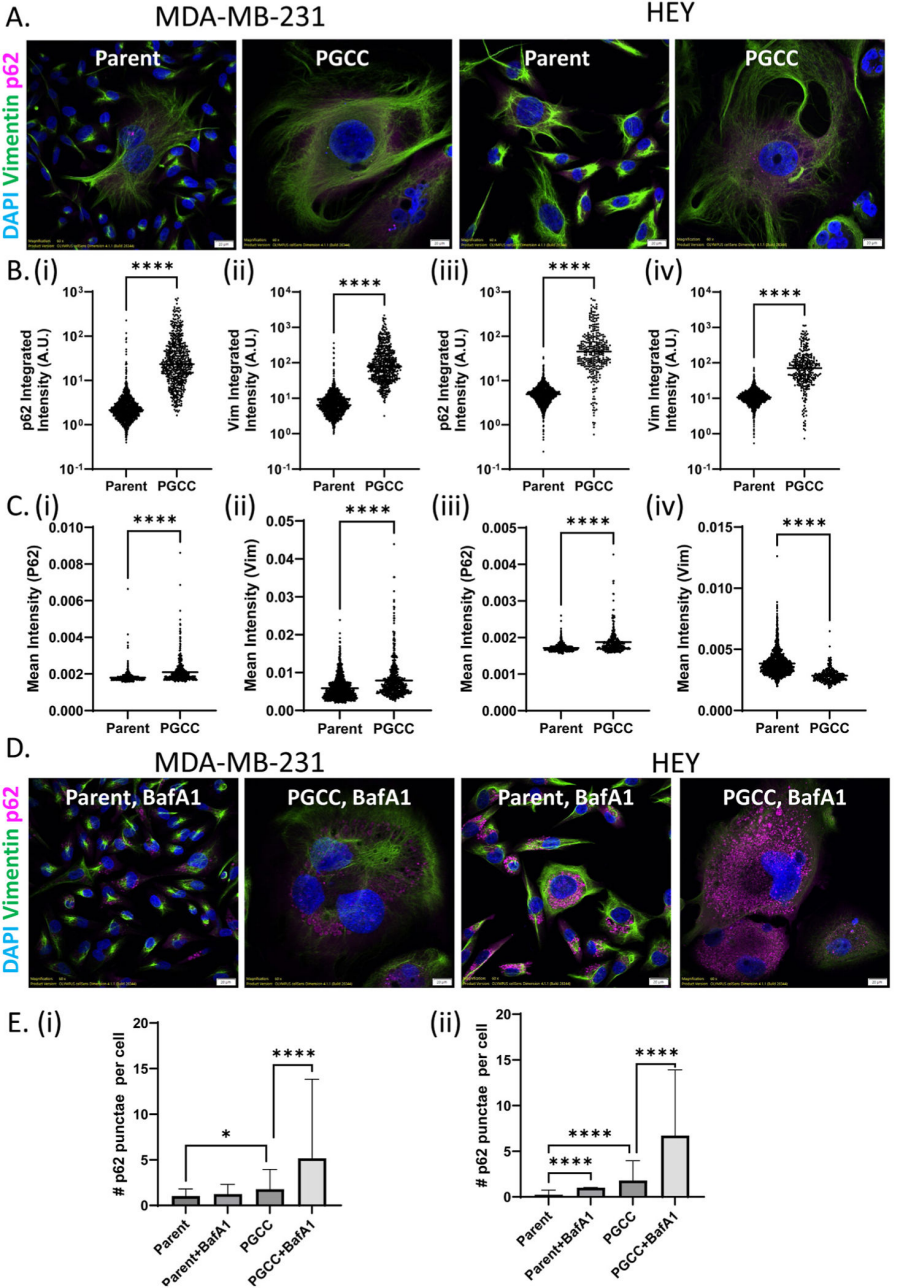
Increased accumulation of P62 after autophagy inhibition in PGCCs. Immunofluorescence staining was used to assess the expression of P62 (magenta) and Vimentin (green). (A) MDA-MB-231 and HEY cells exhibited distinct P62 puncta and demonstrated prominent perinuclear vimentin organization. In contrast, PGCCs showed a more diffuse distribution of both proteins. Representative images were taken with Olympus Yokogawa spinning disk confocal microscope at 60x magnification (Scale = 20um). (B) Quantitative analysis of integrated fluorescence intensity per cell from epifluorescence images (10x magnification) indicated significantly elevated P62 (B-i, iii) and vimentin (B-ii, iv) expression in PGCCs relative to parent cells. (C) Intensity normalized to cell area – mean intensity, was also higher for P62 (C-i, iii). Mean intensity of vimentin was higher in MDA-MB-231 PGCCs but lower in HEY PGCCs compared to parent cells. Statistical analysis was performed on n > 300 cells per group. An unpaired *t*-test with Welch’s correction was applied, and statistical significance was indicated by (****) for p < 0.0001. (D) To assess enhanced autophagic flux, cells were exposed to the autophagy inhibitor Bafilomycin A1 (BafA1) (100 nM) for 24 h, followed by staining for P62 and vimentin. Representative images were taken with Olympus Yokogawa spinning disk confocal microscope at 60x (Scale = 20um). (E) Quantitative analysis of integrated fluorescence intensity per cell from epifluorescence images (10x magnification) of P62 puncta revealed increased accumulation of P62 in MDA-MB-231 PGCCs, but not in parent cells, following BafA1 treatment (E-i). In HEY cells, treatment with BafA1 led to a marked increase in P62 puncta in both parent cells and PGCCs; notably, the elevation was more substantial in PGCCs (E-ii). Bar graph displays mean ± S.D. for n > 300 cells per group. One-way ANOVA followed by Tukey’s post-hoc test was employed to assess group differences, with statistical significance denoted as (*) for p < 0.05 and (****) for p < 0.0001. (For interpretation of the references to colour in this figure legend, the reader is referred to the Web version of this article.)

PGCCs exhibit unique adaptations in their vimentin intermediate filament (VIF) network that support their enlarged morphology and persistent migration [25,32]. Vimentin polarization in cells could be critical in directing autophagic flux for ATP-dependent processes in cells. VIFs are also linked to nuclear lamina [33,34], so this link between VIF structure and autophagic flux may also be critical in eliminating DNA damage. To further examine the role of the vimentin network in regulating autophagy, we assessed p62 (SQSTM1) localization and expression in parent cancer cells and PGCCs. p62 is a selective autophagy substrate that localizes to autophagosomes and is degraded during autophagic flux [35]. Immunofluorescence staining revealed p62 puncta in both parent cells and PGCCs, with visibly higher basal levels in PGCCs (Fig. 4A). Quantification of integrated p62 intensity per cell in CellProfiler confirmed significantly higher p62 expression in PGCCs for both MDA-MB-231 and HEY lines (Fig. 4B–i, iii). Similarly, integrated vimentin intensity was elevated in PGCCs compared to parent cells (Fig. 4B–ii, iv). After normalization to cell area to account for the enlarged size of PGCCs, p62 expression remained significantly higher in PGCCs, whereas vimentin displayed cell line specific differences (Fig. 4C). Specifically, mean vimentin intensity increased in MDA-MB-231 PGCCs but decreased in HEY PGCCs relative to their respective parent cells (Fig. 4C–ii, iv).

To probe autophagic flux, cells were treated with Bafilomycin A1 (BafA1), an inhibitor of autophagosome–lysosome fusion, which leads to p62 accumulation. BafA1 treatment increased p62 puncta in the perinuclear region in both cell types (Fig. 4D). In MDA-MB-231 cells, this increase was significant only in PGCCs, whereas in HEY cells, p62 accumulation was significant in both parent cells and PGCCs, with a greater magnitude of change in PGCCs (Fig. 4E). These results suggest that PGCCs have higher basal autophagic flux compared to parent cells.

**Fig. 5.**
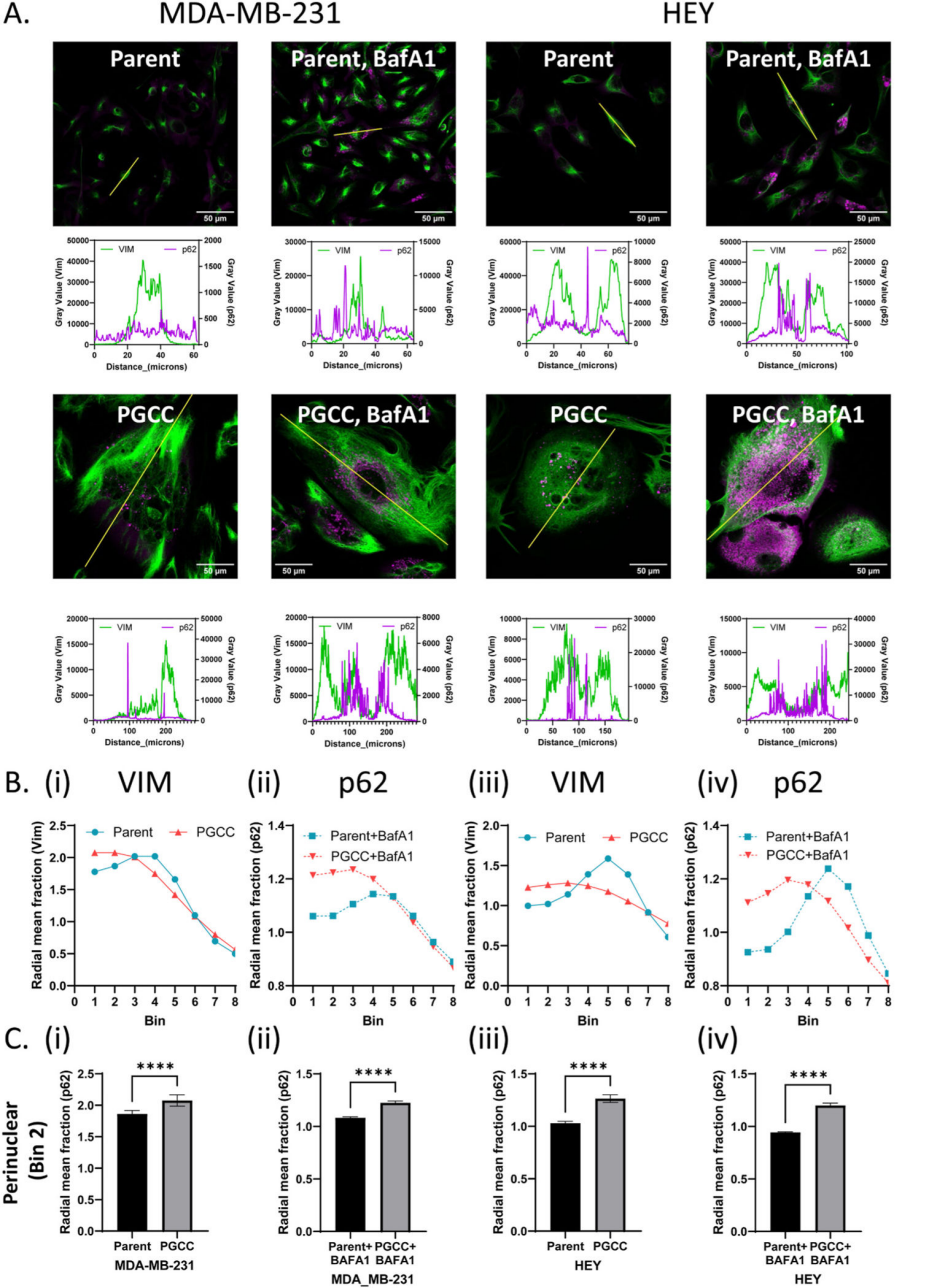
Intracellular distribution of P62 and vimentin was altered in PGCCs. The spatial distribution of each marker was analyzed in individual cells using CellProfiler. (A) Representative immunofluorescence images and corresponding line-scan analysis (yellow line) illustrating P62 (magenta) and vimentin (green) distribution in MDA-MB-231 and HEY cells. Images were acquired with an Olympus Yokogawa spinning disk confocal microscope with 60×-magnification (Scale = 50um). (B-C) Intracellular intensity distribution was quantified by dividing each cell into concentric radial bins extending from the center of the nucleus (bin 1) to the cell periphery (bin 8). The fractional intensity within each bin was calculated and normalized to the fractional pixel area per bin. Radial distribution analysis of vimentin (Vim) revealed the highest fractional intensity in the semi-peripheral region for parent cells and in the nuclear region for PGCCs in both MDA-MB-231 (i) and HEY (iii) cells. HEY PGCCS (iii) displayed a more uniform vimentin distribution across radial bins compared to MDA-MB-231 PGCCs (i). Radial distribution of P62 following 24-h treatment with 100 nM Bafilomycin A1 (BafA1) demonstrated increased perinuclear intensity (bins 1–3) in PGCCs compared to parent cells for both MDA-MB-231 (ii) and HEY cells (iv). (C) Quantification of the mean radial fraction within the near-nuclear region (bin 2) confirmed increased localization of both vimentin and p62 in PGCCs compared to parent cells. Bar graphs display mean ± 95% C.I. Statistical significance was determined using an unpaired *t*-test with Welch’s correction (****for p < 0.0001). (For interpretation of the references to colour in this figure legend, the reader is referred to the Web version of this article.)

To characterize the subcellular distribution of vimentin and p62 in enlarged PGCCs, we performed line-scan analysis in FIJI (Fig. 5A, Supplementary Fig. 9). In parent cells, vimentin intensity profiles were typically uni- or bi-modal with prominent peaks near the perinuclear region, consistent with bundled and condensed vimentin intermediate filaments (VIFs). In contrast, PGCCs exhibited more heterogeneous and dispersed vimentin organization, with lower peak intensities reflecting reduced bundling and broader cytoplasmic—and in some cases nuclear—distribution (notably in HEY PGCCs). For p62, PGCCs displayed narrow but pronounced intensity peaks corresponding to punctate structures, which became more diffusely distributed following BafA1 treatment. We also observed partial overlap between vimentin and p62 intensity peaks in PGCCs, suggesting spatial association. To further quantify these patterns, we performed radial distribution analysis using CellProfiler. Cell areas were divided into eight concentric bins extending from the nuclear center (bin 1) to the cell periphery (bin 8), and fluorescence intensity within each bin was normalized to pixel area (Fig. 5B). In PGCCs, vimentin and p62 intensity fractions were enriched in the nuclear and perinuclear regions (bins 1–3), whereas parent cells showed peak intensity in more medial regions of the cytoplasm. Notably, vimentin distribution differed between cancer types: MDA-MB-231 PGCCs showed strongest enrichment near the nucleus, while HEY PGCCs exhibited a more uniform distribution across the cell interior.

### 3.5. PGCCs display enhanced autophagy to maintain nuclear integrity

**Fig. 6.**
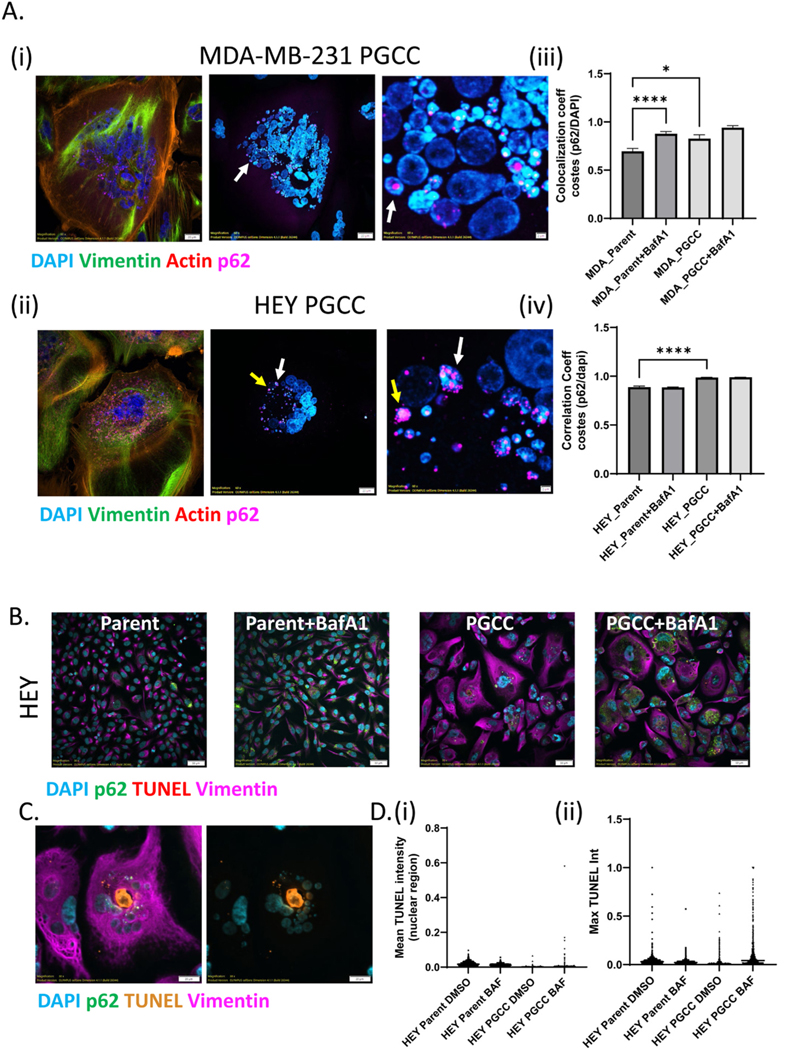
P62 colocalized with fragmented micronuclei in PGCCs. (A) Confocal images show P62 puncta (magenta) inside fragmented micronuclei (blue) in both MDA-MB-231 (i) and HEY (i) PGCCs (scale bar = 20um). Right panel shows a zoomed in region of fragmented PGCC nuclei to highlight colocalization with P62. Colocalization analysis in CellProfiler revealed higher coefficients for P62 over DAPI in PGCCs compared to parent cells for both MDA-MB-231 (iii) and HEY (iv). Bar graphs show mean ± S.E.M. One-way ANOVA followed by Tukey’s post-hoc test was employed to assess group differences, with statistical significance denoted as (*) for p < 0.05 and (****) for p < 0.0001. (B) HEY parent and PGCCs treated with vehicle control or BafA1 were stained for nuclei (blue), p62 (green), TUNEL (red) and vimentin (magenta). Scale Bars = 50um. (C) Zoomed images of TUNEL-positive cells highlight micronuclear localization. (D) We measured mean TUNEL intensity in nuclei, calculated as integrated TUNEL intensity normalized to nuclear area, to identify TUNEL-positive cells (i). To identify apoptotic bodies in the cell, maximum TUNEL intensity (ii) within each cell boundary was quantified. (For interpretation of the references to colour in this figure legend, the reader is referred to the Web version of this article.)

The prominent perinuclear localization of p62 in PGCCs prompted us to examine its association with nuclear structures. Immunofluorescence analysis revealed frequent colocalization of p62 with micronuclei in PGCCs, suggesting engagement of the autophagic pathway in micronuclear turnover (Fig. 6A). Quantification of p62–DAPI colocalization using Costes correlation coefficient confirmed significantly higher colocalization in PGCCs compared to parent cells (Fig. 6A). In parent cells, correlation coefficients indicated moderate (~0.7) and strong (~0.89) association in MDA-MB-231 and HEY parent cells, respectively. In contrast, PGCCs exhibited strong (~0.83) and very strong (~0.99) colocalization in MDA-MB-231 and HEY cells, respectively. To determine whether nuclear fragmentation reflected apoptotic cell death, we performed TUNEL staining in HEY cells (Fig. 6B). Only a small fraction of cells exhibited TUNEL-positive nuclear staining (Fig. 6C–D). While discrete nuclear fragments within PGCCs were occasionally TUNEL-positive and corresponded to regions of high maximum TUNEL-intensity (Fig. 6D), live-cell imaging confirmed that these PGCCs remained viable and functionally active (Supplementary Video 1).

**Fig. 7.**
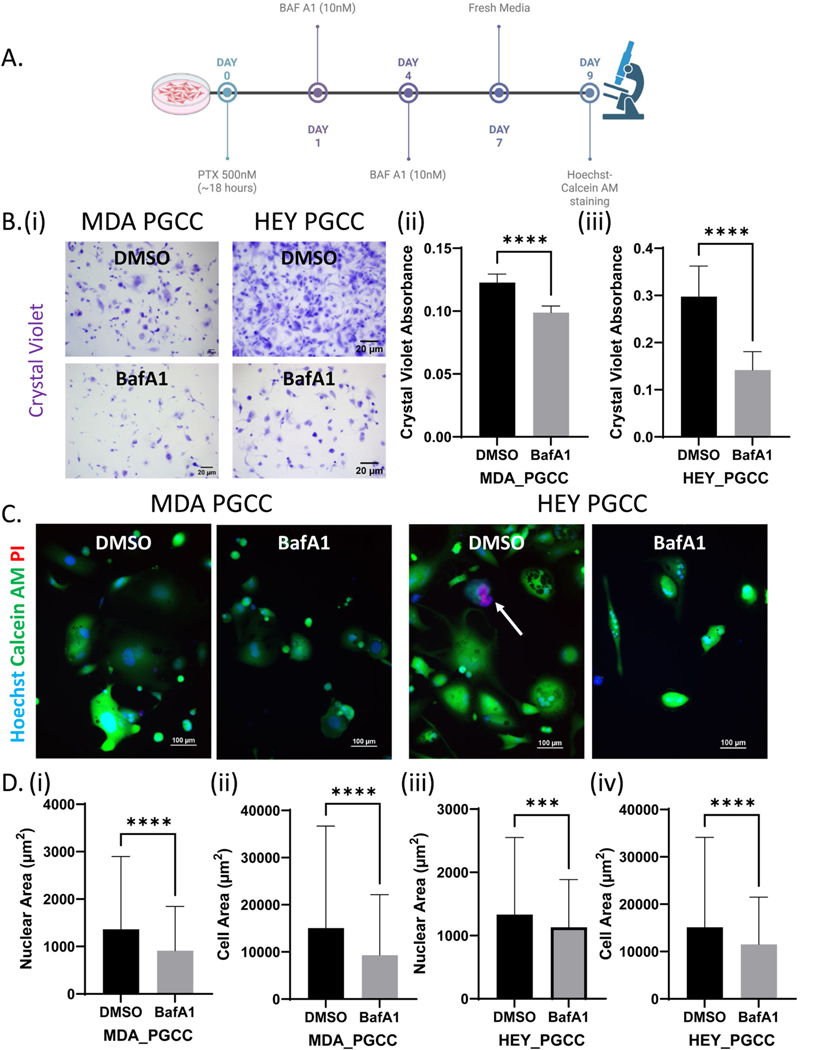
Targeting autophagy reduces PGCC survival and limits PGCC size during chemotherapy. (A) MDA-MB-231 and HEY cells were treated with 500 nM paclitaxel (PTX) for 18 h, followed by an 8-day recovery with either vehicle control or 10 nM Bafilomycin A1 (BafA1) treatment (2x) before measuring nuclear and cell size. A schematic of the treatment schedule is shown. (B) Cells were fixed and stained with crystal violet to assess cell viability (Scale bars = 20um). BafA1 treatment reduced the number of PGCCs for both MDA-MB-231(ii) and HEY cells (iii). Statistical analysis was performed using an unpaired *t*-test with Welch’s correction (N = 2 experiments with n ≥ 3 replicates per condition) with significance indicated as (****) for p < 0.0001. (C-D) Cells were stained with Hoechst 33342, Calcein AM and Propidium Iodide (PI) to identify live cells using fluorescence microscopy (Scale = 100um). (D) Nuclear and cell areas were significantly reduced for MDA-MB-231 (ii, iii) and HEY-PGCCs (iv, v). Statistical analysis was performed using an unpaired *t*-test with Welch’s correction (N = 3, n > 450 cells per condition), with significance indicated as (***) for p < 0.001 and (****) for p < 0.0001. (For interpretation of the references to colour in this figure legend, the reader is referred to the Web version of this article.)

To assess the long-term impact of inhibiting autophagy on nuclear size and integrity, PGCCs were treated with BafA1 for 9 days (Fig. 7A). This treatment led to significant reduction in PGCC number after 9 days (Fig. 7B). Chronic inhibition of autophagic flux reduced the average nuclear and cell areas of PGCCs (Fig. 7C–D; Supplementary Fig. 6) and decreased the number of distinct nuclear structures, including micronuclei (Supplementary Fig. 6A). These changes likely reflect apoptosis of larger PGCCs with accumulated nuclear damage in the absence of efficient autophagic clearance. In addition, cell and nuclear size heterogeneity, measured by CV, was lower after prolonged BafA1 treatment (Supplementary Fig. 6B). Together, these findings indicate that autophagy can play a significant role in maintaining nuclear integrity, supporting DNA synthesis, and sustaining the enlarged nuclear size characteristic of PGCCs.

### 3.6. PGCCs display autophagy-dependent migration

**Fig. 8.**
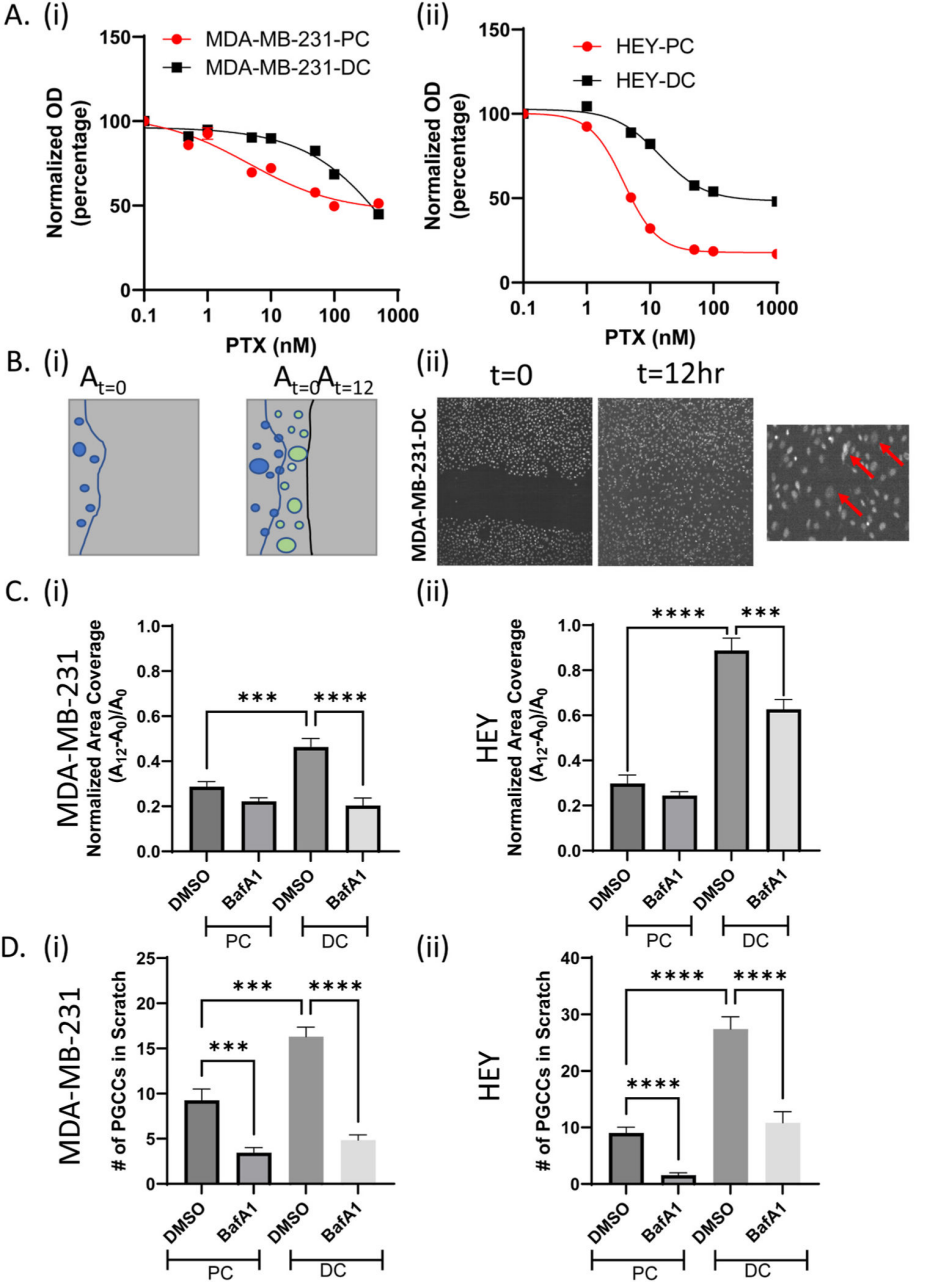
Autophagy drives directional PGCC migration. (A) MTT viability assays were performed to assess the responsiveness of PGCC-derived daughter cells (DCs) to 72-h paclitaxel (PTX) treatment. MDA-MB-231(i) and HEY (ii) DCs demonstrated higher resistance to PTX relative to the parent cells (PCs). (B) Scratch wound assays were conducted to quantify migratory capacity of DCs. A uniform scratch was introduced into a confluent monolayer at t = 0, and wound closure was measured after 12 h (t = 12) as outlined in the schematic (i). Representative images of Hoechst 33342-stained MDA-MB-231 DCs at t = 0 and t = 12 h are shown. Red arrows indicate migrated PGCCs. (C) Quantification of wound closure at 12 h was normalized to the initial wound area with or without Bafilomycin A1 (BafA1) treatment for PCs and DCs. MDA-MB-231 (i) and HEY (i) DCs showed increased wound closure compared to PCs. BafA1 treatment resulted in significantly reduced wound closure for MDA-MB-231 DCs. (D) Quantification of PGCCs within the wound area revealed reduced numbers in both PCs and DCs following BAfA1 treatment (i). DCs showed a higher number of PGCCs within the wound region compared to PCs (ii). Bar graphs show mean ± S.E.M. One-way ANOVA followed by Tukey’s post-hoc test was employed to assess group differences (N = 2), with statistical significance denoted as (***) for p < 0.01, and (****) for p < 0.0001. (For interpretation of the references to colour in this figure legend, the reader is referred to the Web version of this article.)

PGCCs are a dormant subpopulation capable of generating new tumor cells through amitotic budding. To assess whether their progeny retains drug resistance, we isolated daughter cell (DC) clones from PTX-induced PGCCs in both MDA-MB-231 and HEY cell lines. These DC clones were heterogeneous but consistently showed greater resistance to PTX compared to their respective parent cells (Fig. 8A, Supplementary Fig. 7).

We next examined the role of autophagy in cell migration using a scratch wound assay (Fig. 8B). The MDA-MB-231 and HEY DC clones demonstrated greater scratch wound area closure than their parent cells, with increases of ~1.5-fold (p < 0.001) and ~3-fold (p < 0.0001), respectively (see Fig. 8C). In MDA-MB-231 and HEY DC clones, administration of BafA1 led to a significant reduction in the area occupied by migrating cells—by 57% (p < 0.0001) and 29% (p < 0.001), respectively—after 12 h, relative to untreated controls (Fig. 8C). Similar trends were observed in parent cells although these differences were not statistically significant.

We also quantified the migration of PGCCs into the scratch wound area, observing that most PGCCs exhibited directional movement indicative of active migration, whereas a subset remained stationary (Supplemental Videos 2–4). PGCCs in the scratch wound increased by 1.7 times in MDA-MB-231 DCs (p < 0.001) and 3 times in HEY DCs (p < 0.0001) compared to their parent cells. BafA1 significantly reduced PGCC migration into wound scratch areas for both parent cells and DCs. These findings suggest that autophagy contributes to the migratory capacity of PGCC-derived daughter cells. Furthermore, PGCCs in DCs that have recovered from treatment are dependent on autophagy same as parent cells.

## 4. Discussion

Polyploid giant cancer cells (PGCCs) remain a formidable obstacle in the treatment of TNBC and OC due to their intrinsic chemoresistance and ability to repopulate tumors through amitotic budding. In this study, we identify autophagy as a critical survival and functional pathway in PTX-induced PGCCs, with a particular role in maintaining nuclear integrity and supporting migration.

We first confirmed that PTX-induced PGCCs in both MDA-MB-231 and HEY cells are substantially larger, more heterogeneous, and highly resistant to subsequent PTX exposure (Fig. 1, Supplementary Fig. 1). PTX treatment significantly increased PGCC size compared to the naive population: MDA-MB-231 PGCCs grew from 6777 μm^2^ to 22,465 μm^2^ (>3-fold), and HEY PGCCs from 3426 μm^2^ to 15,383 μm^2^ (>4-fold). We also identified PGCCs in TNBC and HGSOC samples that were heterogeneous in both size and frequency across different stages of tumor samples (Fig. 1 and Supplementary Fig. 2). In the analyzed tissue microarrays, TNBC and HGSOC cases were primarily stage II/III, with limited representation of stage IV tumors, likely due to prior surgery, chemotherapy exposure, and metastatic dissemination reducing availability of primary tumor tissue. Stratification by chemotherapy exposure would improve interpretation, given its established role in promoting PGCC formation. However, treatment history was not provided for these specimens, precluding subgroup analysis. Future studies using clinically annotated cohorts will be necessary to directly assess the impact of prior therapy on PGCC abundance.

Transcriptomic profiling revealed broad gene expression changes, with significant enrichment of metabolic and cytoskeletal pathways and strong upregulation of autophagy-related genes, including *SQSTM1 (P62)* and *DRAM1*. These findings are consistent with prior reports demonstrating that autophagy plays a critical role in PGCC dormancy and survival [36,37]. Roh et al. previously found that PGCCs contain increased mitochondrial mass, resulting in elevated ROS production [38]. You et al. demonstrated that chemotherapy induces mitochondrial structural damage that is subsequently resolved through mitophagy [36]. More recently, Bona et al. and Martin et al. independently demonstrated that mitochondria-derived ROS can damage micronuclei and recruit p62/SQSTM1 [39,40]. In agreement with these studies, we observed elevated oxidative stress in PGCCs, characterized by increased ROS levels and strong upregulation of mitochondrial superoxide dismutase 2 (SOD2), suggesting enhanced mitochondrial ROS buffering as a key adaptive response (Fig. 3). We also found frequent localization of p62 to micronuclei in PGCCs (Figs. 5–6). Although Martin et al. did not observe evidence of canonical autophagy-mediated micronuclear degradation, we identified instances in which smaller nuclei appeared enclosed within p62-positive structures resembling autophagosomes. Importantly, chronic inhibition of autophagic flux using BafA1 reduced both cell and nuclear size and decreased the prevalence of micronuclei. Together, these findings support a role for autophagy in sustaining nuclear architecture under prolonged stress (Fig. 6).

A major structural feature of PGCCs is their dispersed vimentin intermediate filament (VIF) network. Compared to the perinuclear bundling seen in parent cells, PGCC VIFs are more evenly distributed and often polarized at the leading edge, where they can scaffold lysosomes and autophagosomes [26,27]. This organization may facilitate autophagosome–lysosome fusion, spatially coordinate autophagic flux, and channel ATP production toward energy-intensive processes such as persistent migration [26,27,41]. Indeed, BafA1 treatment impaired migration in PGCC-derived daughter cells (Fig. 8). These findings suggest that vimentin-regulated autophagy contributes not only to PGCC survival but also to their invasive potential. Further studies using naive PGCCs will be necessary to determine if vimentin-dependent autophagy plays similar roles in their invasive behavior.

Our work focused on macroautophagy, as assessed by p62 localization and accumulation after autophagic flux inhibition. However, chaperone-mediated autophagy (CMA) may also be active in PGCCs and could compensate when macroautophagy is inhibited [42,43]. Both pathways may work in concert to sustain the high metabolic and structural demands of these cells. Notably, we observed distinct differences between MDA-MB-231 and HEY PGCCs (Supplementary Fig. 8), particularly in the roles of actin and vimentin in compartmentalizing micronuclei that colocalize with p62. However, definitive determination of micronuclear origin and fate will require dynamic imaging or lineage-tracing approaches to conclusively elucidate the underlying mechanism. While both MDA-MB-231 and HEY PGCCs showed elevated autophagy compared to parent cells, differences in nuclear autophagy patterns and migration dependence suggest cell line–specific utilization of unique cytoskeleton dependent autophagy pathways. Furthermore, it will be important to investigate the coordinated roles of cytoskeletal proteins in amitotic budding, drawing parallels to actin-mediated budding in yeast, and to determine whether vimentin serves a comparable structural or mechanical function in PGCCs. High-resolution live-cell imaging could further clarify how VIF organization couples to autophagic flux, nuclear remodeling, and budding.

Therapeutically, targeting autophagy in PGCCs represents a promising approach, but several challenges remain [44,45]. Using autophagy inhibition as a cancer treatment may cause serious systemic toxicity in patients. Clinical trials with chloroquine and hydroxychloroquine have produced mixed results—some have shown positive effects, while others have not [46–48].Inhibiting autophagy after PTX treatment reduces PGCC size and number, but this may select for PGCC subpopulations less reliant on macroautophagy or more dependent on CMA. A prior study by Bowers et al. demonstrated that autophagy inhibition alone was insufficient to block PGCC formation under chemotherapeutic stress, although it significantly reduced colony-forming capacity [49]. Future work should compare naïve and PTX-induced PGCCs to determine how induction method influences vimentin structure, autophagy reliance, and stress response pathways. Targeting autophagy in a context dependent manner i.e. vimentin-p62 interaction in PGCCs, may increase therapeutic efficacy and limit toxicity to adjacent tissues.

In summary, our findings reveal that autophagy in PGCCs is intimately linked to oxidative stress management, nuclear maintenance, and migration, and is spatially organized by a distinct vimentin network. By elucidating these mechanisms, we identify potential vulnerabilities in PGCC biology that could be exploited to limit tumor recurrence and chemoresistance in aggressive breast and ovarian cancers.

## Supplementary Material

1

2

3

4

5

6

7

8

9

10

11

12

13

14

15

16

## Data Availability

Study data are available from the corresponding author upon reasonable request.

## Appendix A. Supplementary data

Supplementary data to this article can be found online at https://doi.org/10.1016/j.canlet.2026.218431.
