# Supplementary material for: Autophagy is essential for survival and function of polyploid giant cancer cells under therapeutic stress
Source: Cancer Lett. Author manuscript; Available in PMC 2026 Aug 10. (PMC13455339; doi:10.1016/j.canlet.2026.218431)
Supplement: Supplementary file 2 [file NIHMS2180034-supplement-11.pdf]

Supplementary Figure 3

A.

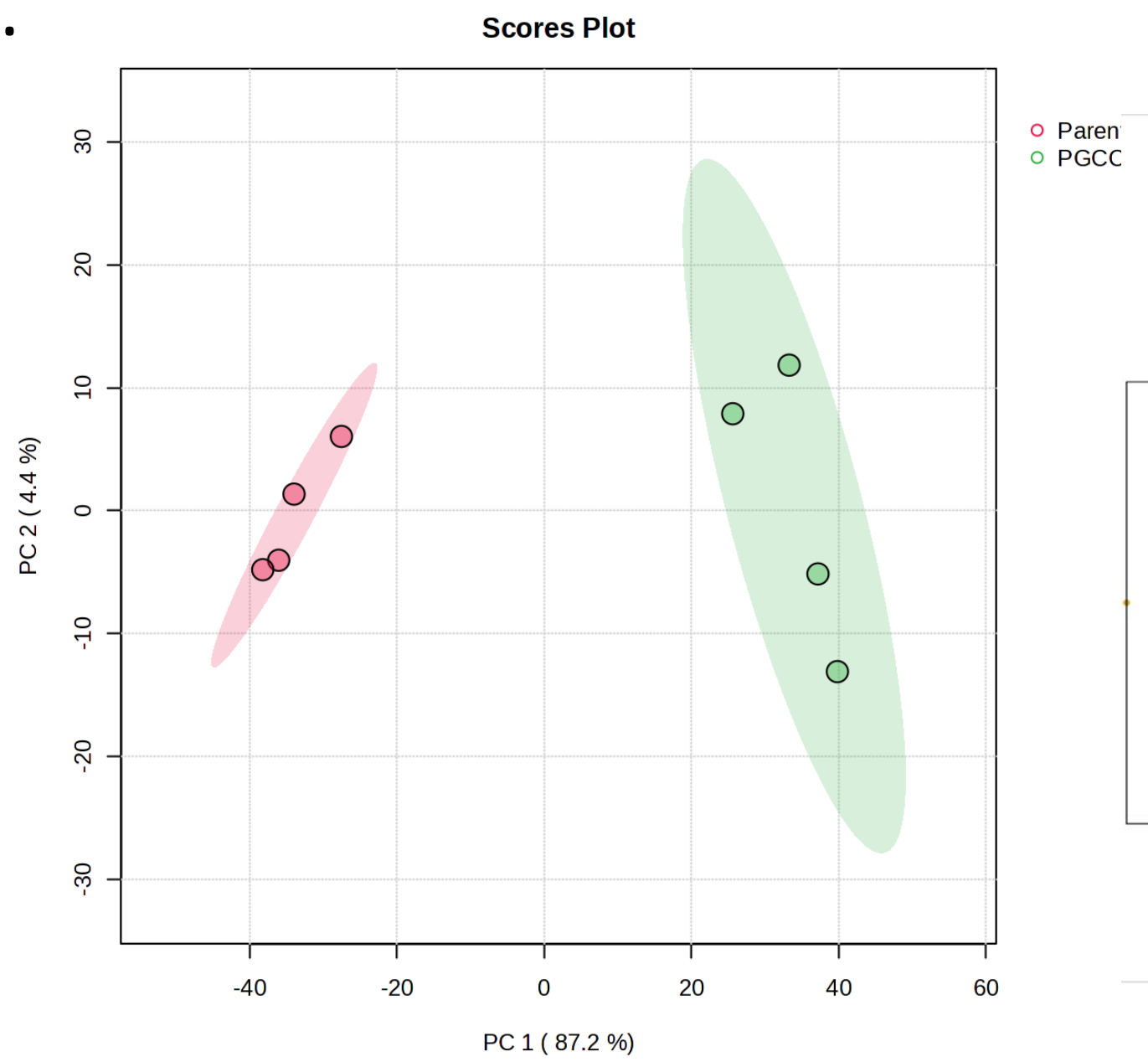

B.

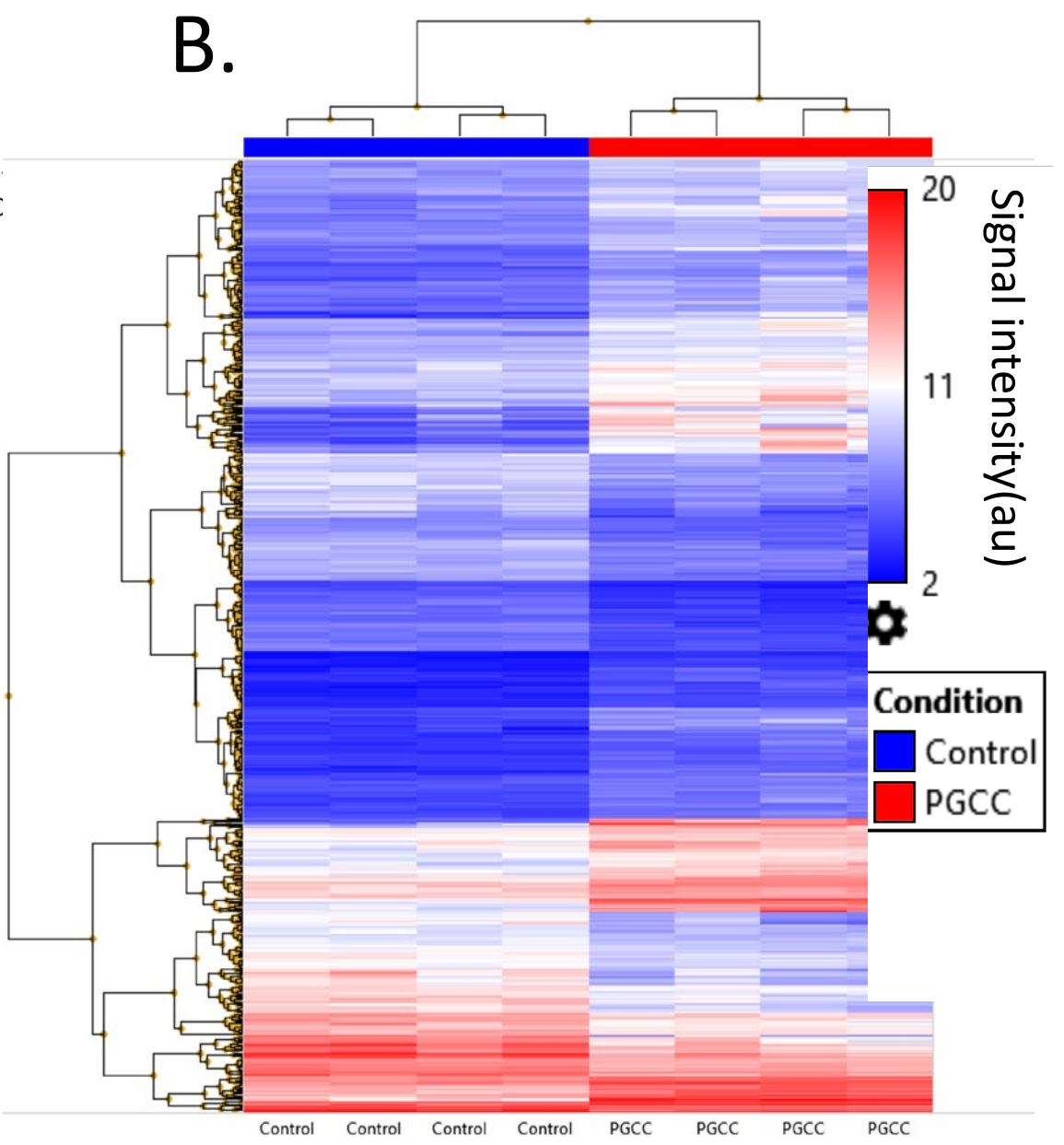

C.

Gene Symbol	Description	Log2FC	P-val	FDR P-val
HIST1H2BM	histone cluster 1, H2bm	-9.67	9.88E-06	0.0170
HIST1H2AG	histone cluster 1, H2ag	-4.76	2.75E-05	0.0268
HIST1H2AJ	histone cluster 1, H2aj	-4.6	1.02E-05	0.0170
CDCA3	cell division cycle associated 3	-4.41	2.33E-05	0.0248
HIST1H2AI	histone cluster 1, H2ai	-4.3	4.61E-05	0.0350
PLK1	polo-like kinase 1	-4.14	4.49E-05	0.0346
MYBL2	v-myb avian myeloblastosis viral oncogene homolog-like 2	-4.11	2.80E-05	0.0269
HIST1H2AM/H3J	histone cluster 1, H2am; histone cluster 1, H3j	-3.93	8.92E-05	0.0488
AURKB	aurora kinase B	-3.9	5.88E-05	0.0390
HIST1H2BB	histone cluster 1, H2bb	-3.87	4.02E-05	0.0321
NCAPD2	non-SMC condensin I complex subunit D2	-3.83	5.25E-05	0.0370
PHF19	PHD finger protein 19	-3.52	1.04E-05	0.0170
FANCA	Fanconi anemia complementation group A	-3.45	1.62E-05	0.0215
HMGN2	high mobility group nucleosomal binding domain 2	-3.42	3.92E-05	0.0316
CDKN2C	cyclin-dependent kinase inhibitor 2C (p18, inhibits CDK4)	-3.33	1.74E-05	0.0220
HIST2H2BF	histone cluster 2, H2bf	-3.29	3.30E-05	0.0296
PSRC1	proline/serine-rich coiled-coil 1	-3.24	4.68E-05	0.0353
INCENP	inner centromere protein	-3.13	8.01E-05	0.0461
HIST1H2BL	histone cluster 1, H2bl	-3.12	3.54E-05	0.0305
SKP2	S-phase kinase-associated protein 2, E3 ubiquitin protein ligase	-3.1	9.93E-06	0.0170
CHAF1B	chromatin assembly factor 1, subunit B (p60)	-3.03	2.11E-05	0.0233
MCM5	minichromosome maintenance complex component 5	-3	1.66E-06	0.0062
MCM3	minichromosome maintenance complex component 3	-2.9	3.78E-05	0.0313
