# Supplementary material for: Autophagy is essential for survival and function of polyploid giant cancer cells under therapeutic stress
Source: Cancer Lett. Author manuscript; Available in PMC 2026 Aug 10. (PMC13455339; doi:10.1016/j.canlet.2026.218431)
Supplement: Supplementary file 3 [file NIHMS2180034-supplement-12.pdf]

Supplementary Figure 4

A.

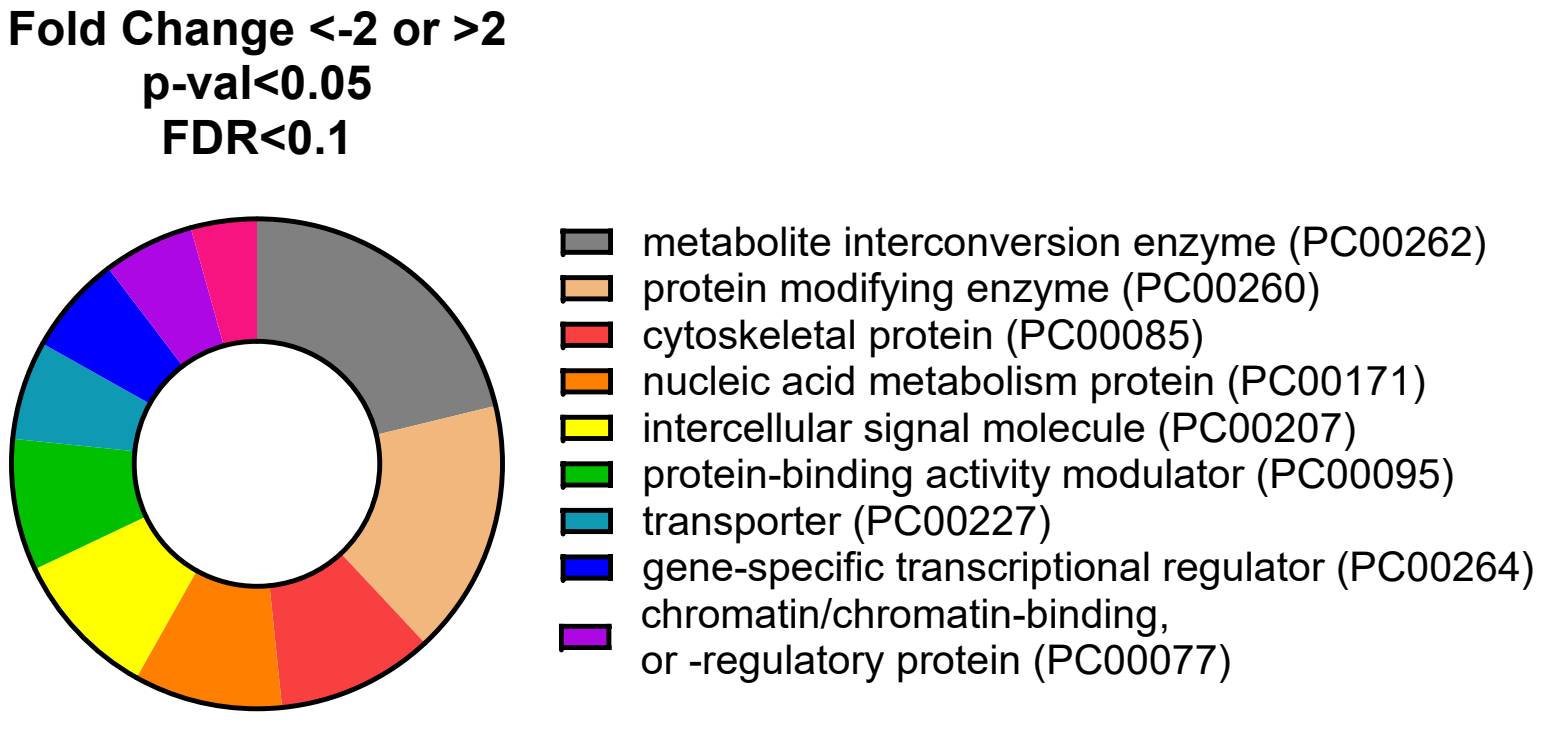

B.

	Activation z-score	-log10(p)
PPAR Signaling	-2.828	3.83
Cell Cycle Control of Chromosomal Replication	-2.449	3.25
Antioxidant Action of Vitamin C	-2.236	2.42
Kinetochore Metaphase Signaling Pathway	-1.89	3.17

C.

	**Log2FC**	
	**MDA-MB-231**	**HEY**
**SOD2**	**4.1**	**2.2**
