# Supplementary material for: Autophagy is essential for survival and function of polyploid giant cancer cells under therapeutic stress
Source: Cancer Lett. Author manuscript; Available in PMC 2026 Aug 10. (PMC13455339; doi:10.1016/j.canlet.2026.218431)

Supplementary Figure 6

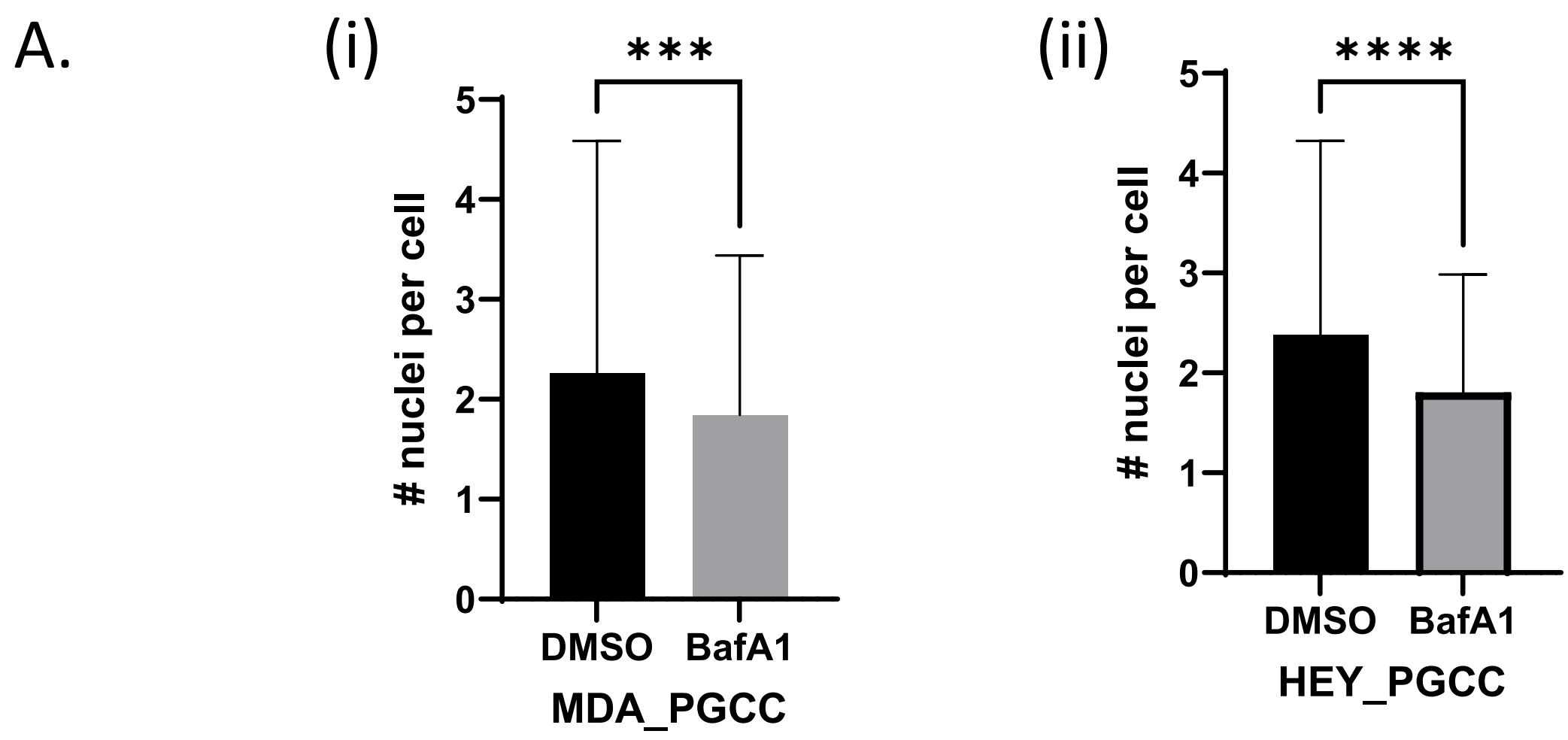

B.

Coeff. Of variation	MDA_ PGCC+DMSO	MDA_ PGCC+BafA1	HEY_ PGCC+DMSO	HEY_ PGCC+BafA1
Nuclear area	113%	102%	91.7%	66.6%
Cell area	144%	138%	126%	86.5%

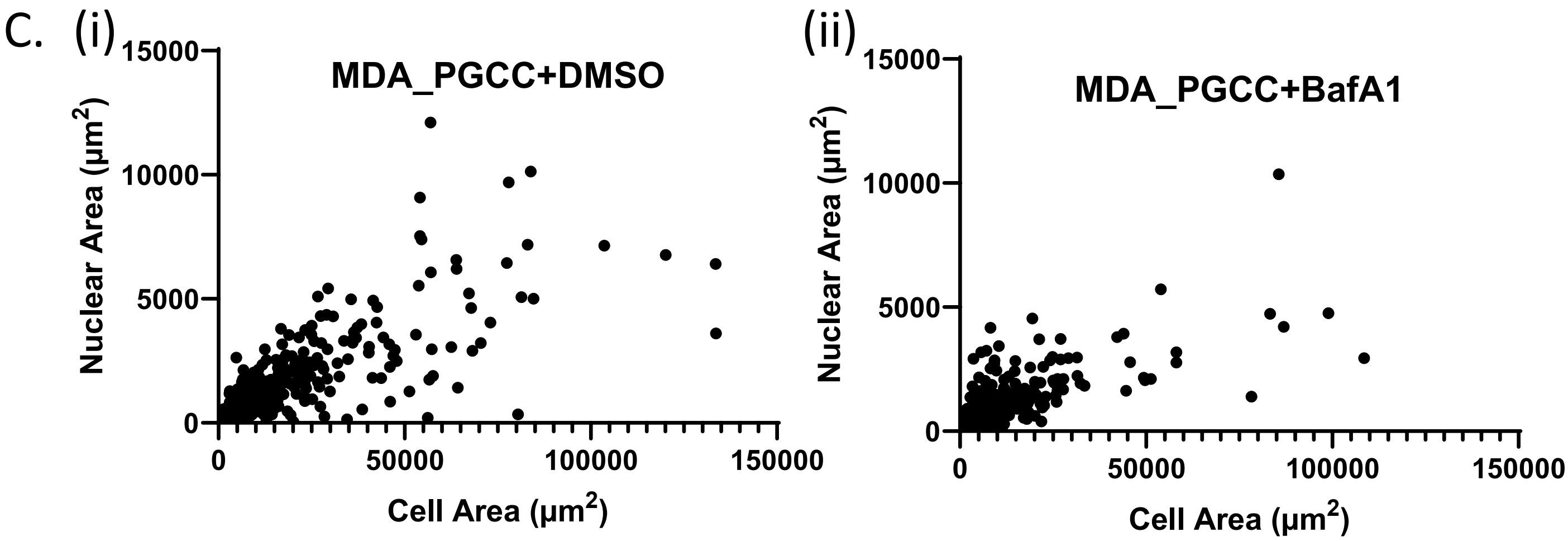

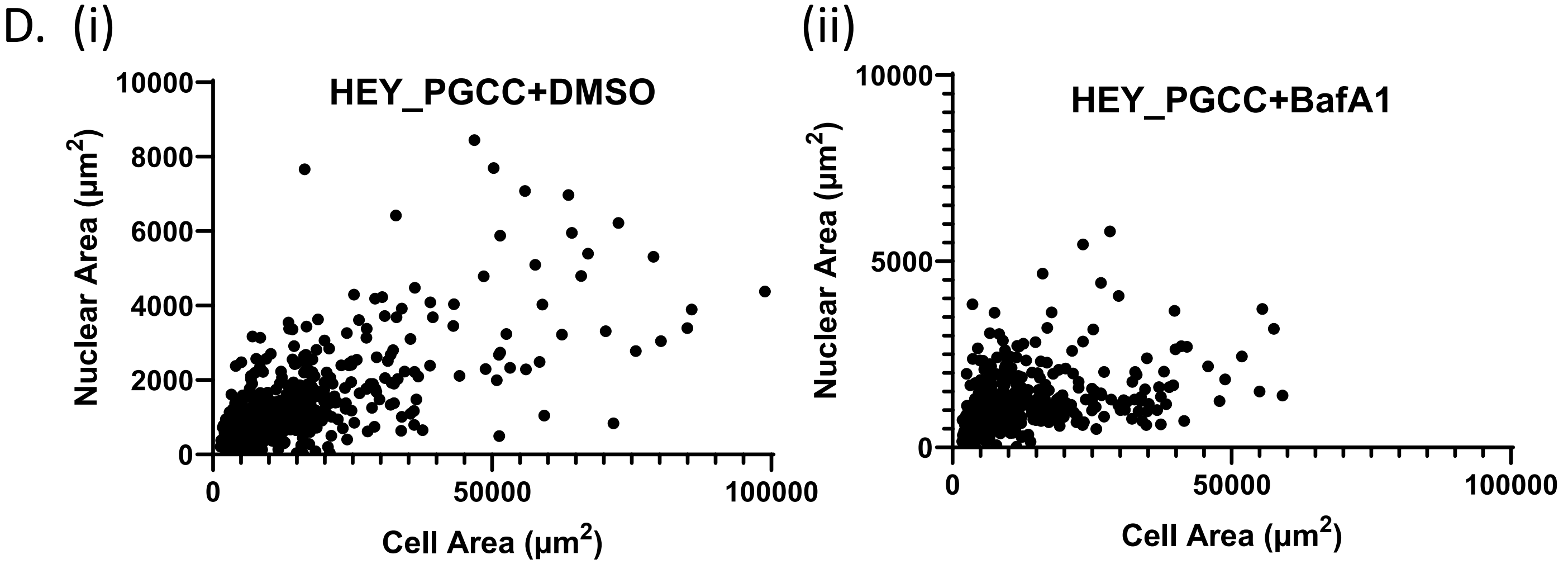

Supplement: Supplementary file 5 [file NIHMS2180034-supplement-14.pdf]
