# Supplementary material for: Autophagy is essential for survival and function of polyploid giant cancer cells under therapeutic stress
Source: Cancer Lett. Author manuscript; Available in PMC 2026 Aug 10. (PMC13455339; doi:10.1016/j.canlet.2026.218431)

Supplementary Figure 7

A.

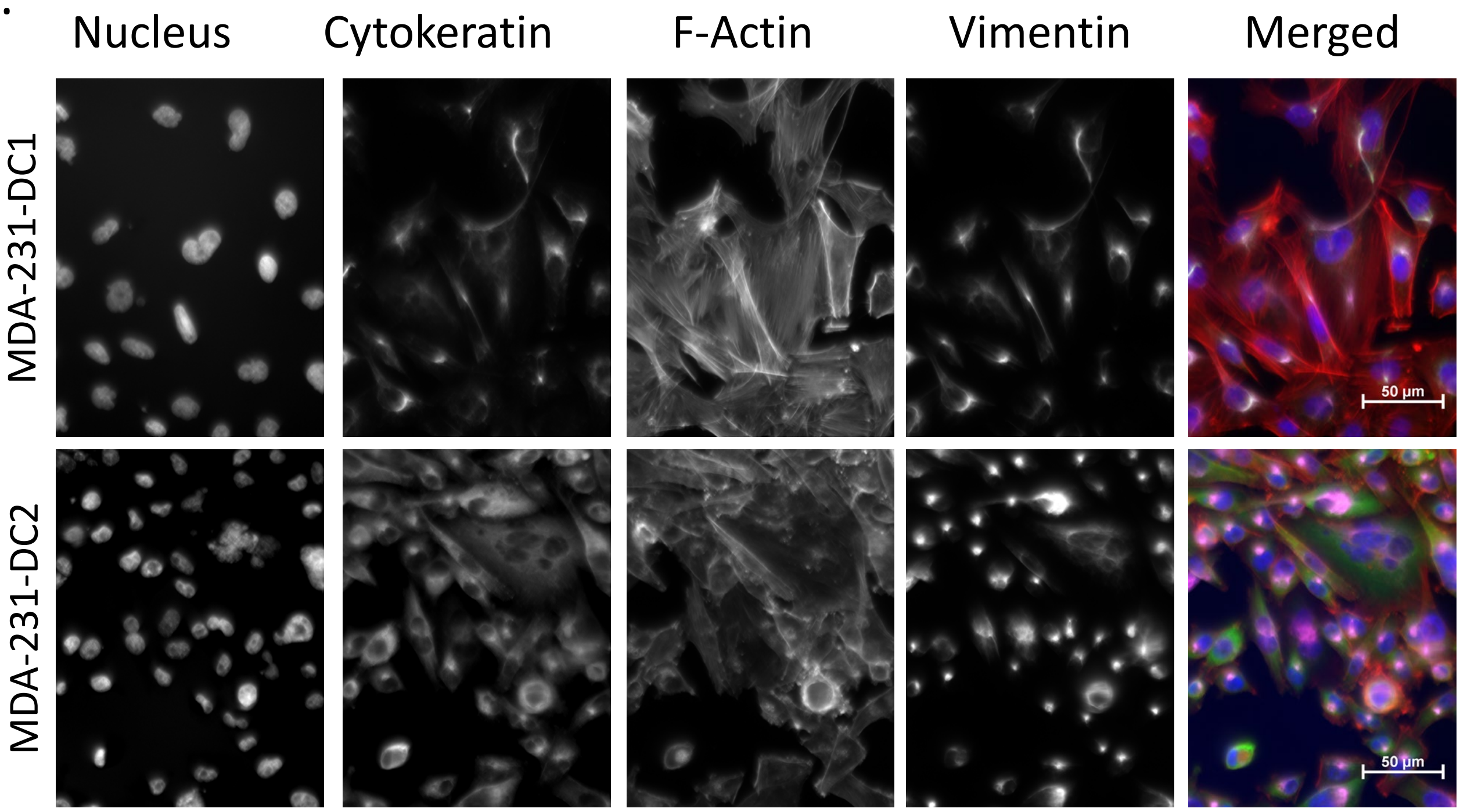

B.

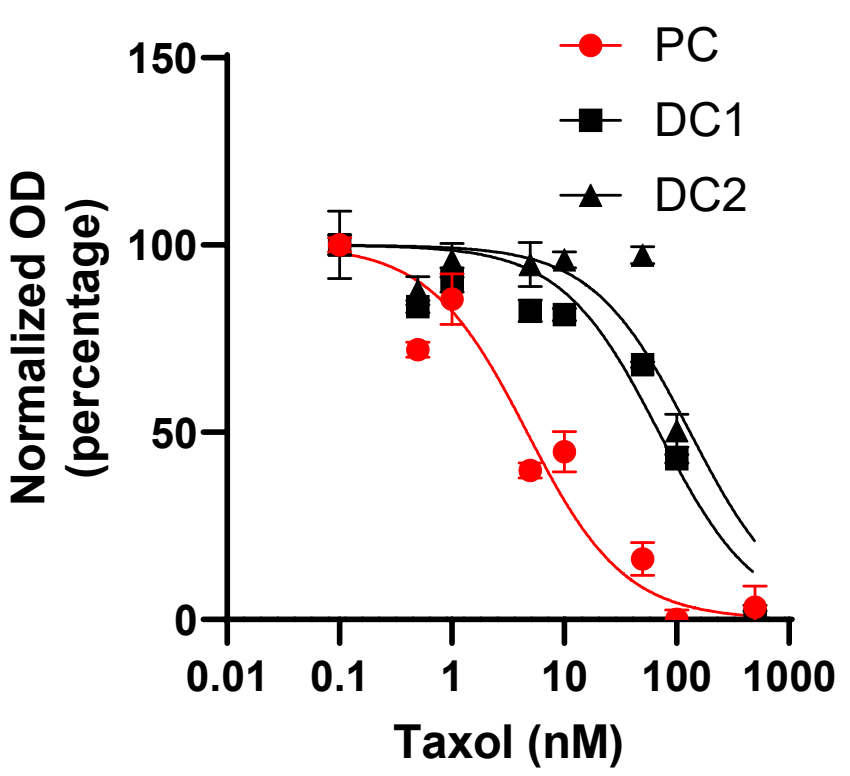

C.

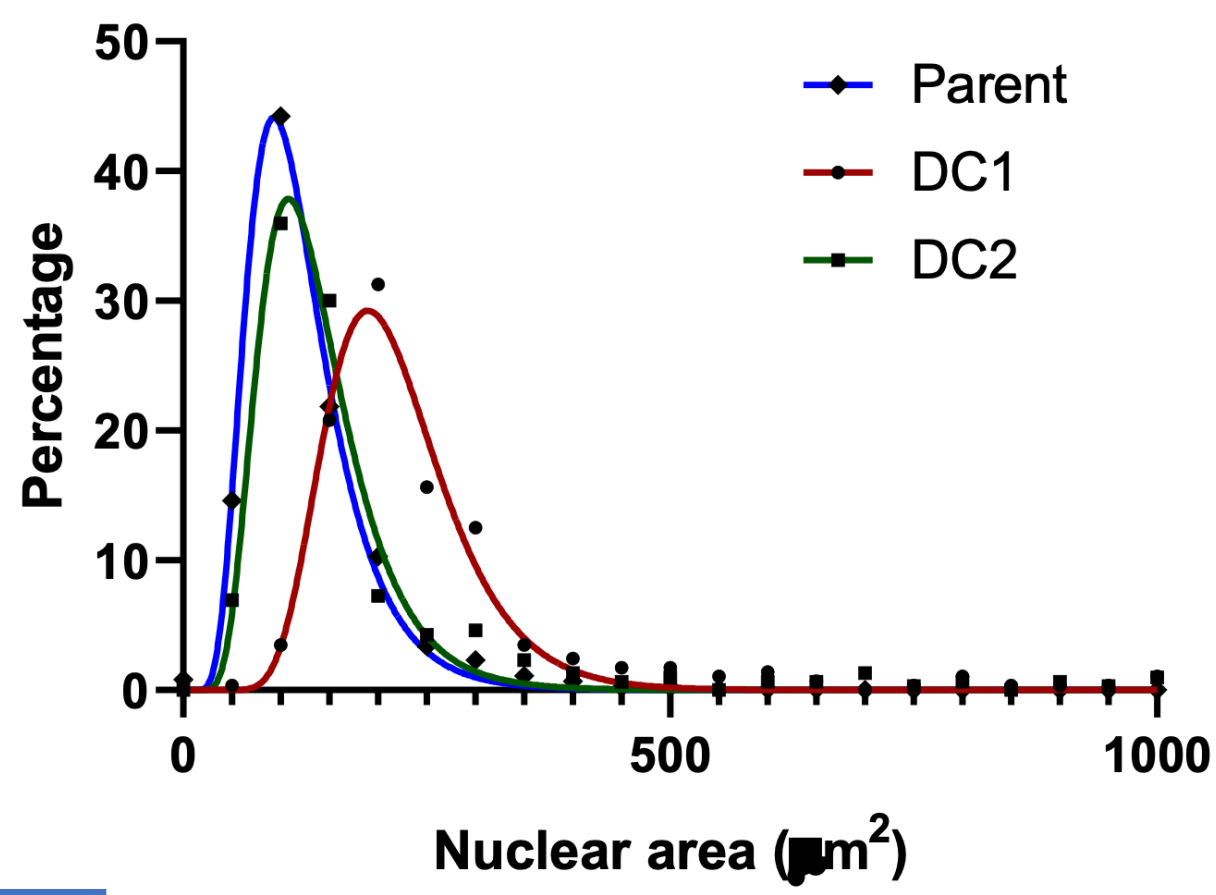

Taxol	PC	DC1	DC 2
IC50 (nM)	4.68	69.67	131.9
95% CI	2.392 to 8.983	36.66 to 130.4	62.34 to 292.7

D. (i)

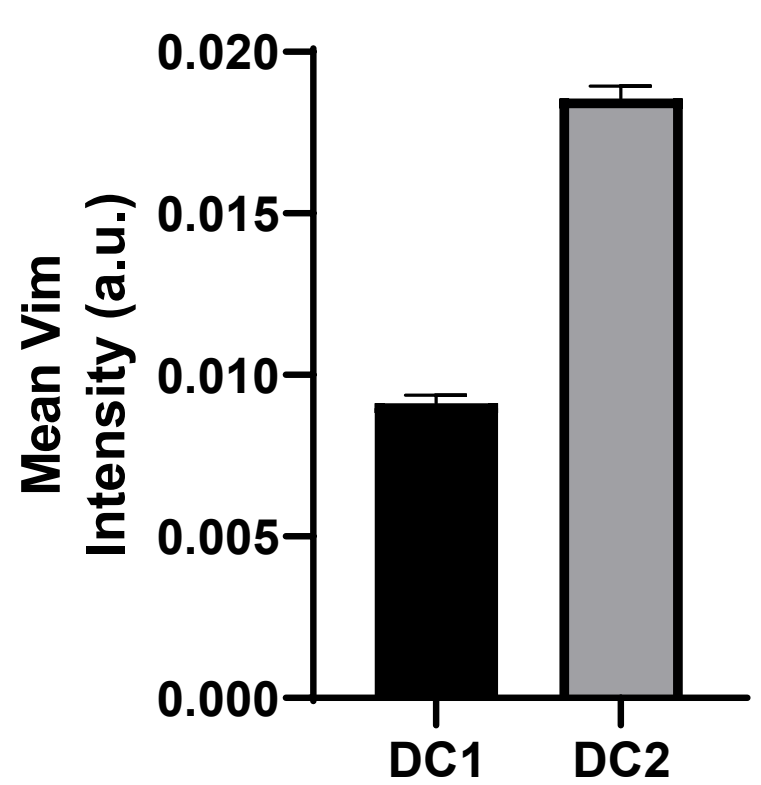

(ii)

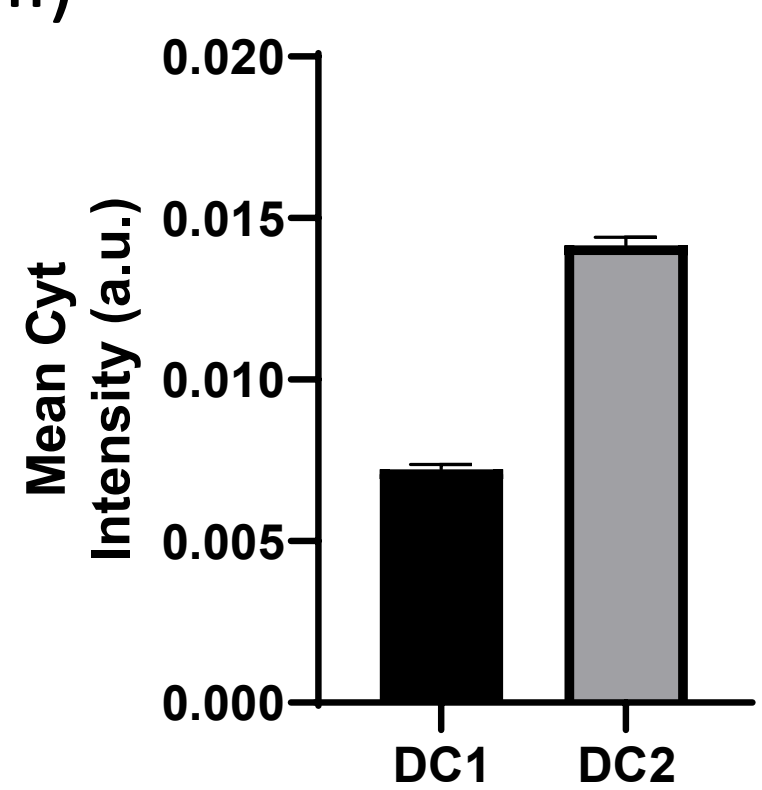

Supplement: Supplementary file 6 [file NIHMS2180034-supplement-15.pdf]
