# Supplementary material for: Autophagy is essential for survival and function of polyploid giant cancer cells under therapeutic stress
Source: Cancer Lett. Author manuscript; Available in PMC 2026 Aug 10. (PMC13455339; doi:10.1016/j.canlet.2026.218431)

Supplementary Figure 9

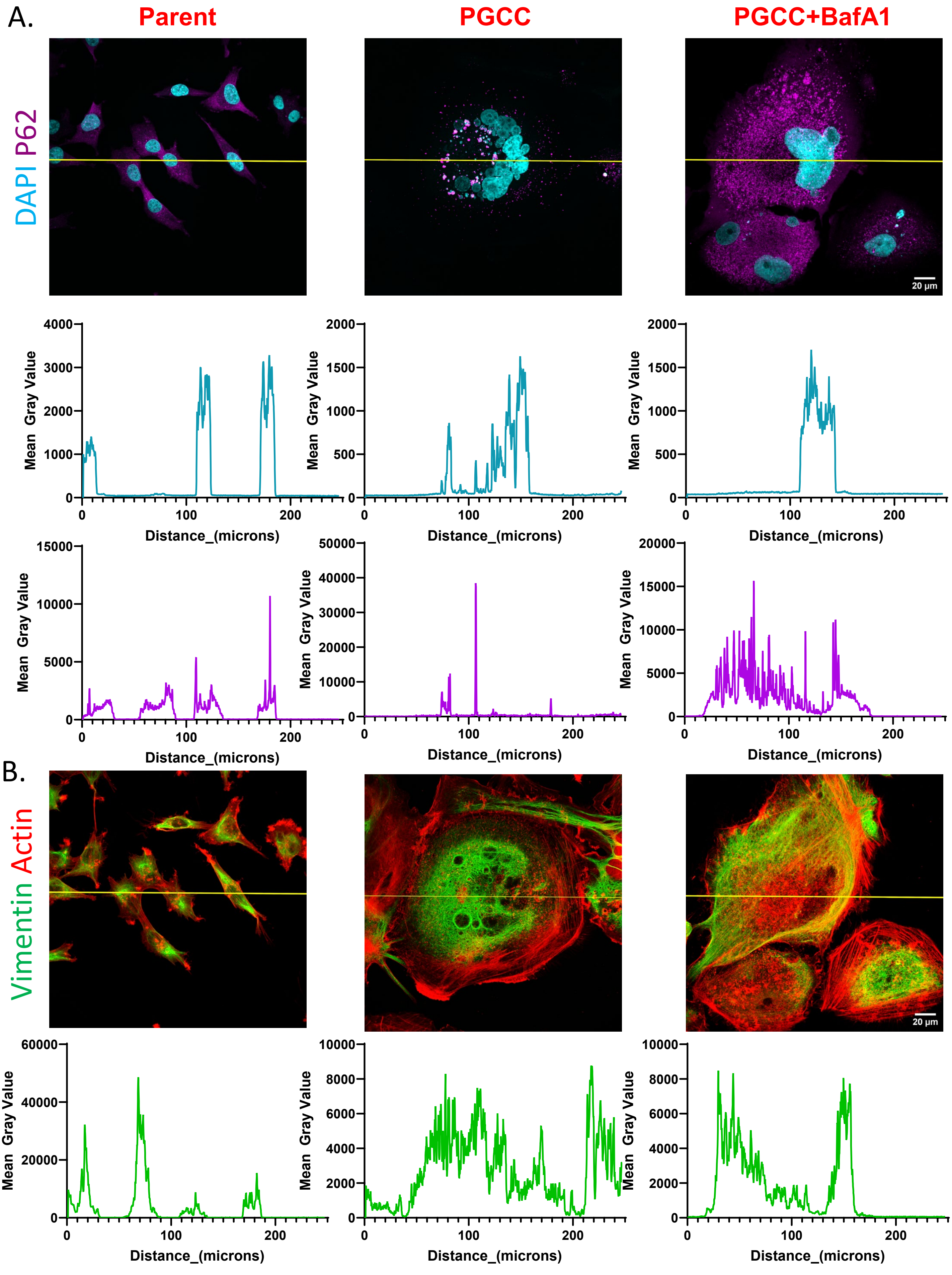

Supplement: Supplementary file 8 [file NIHMS2180034-supplement-2.pdf]
