# Supplementary material for: Autophagy is essential for survival and function of polyploid giant cancer cells under therapeutic stress
Source: Cancer Lett. Author manuscript; Available in PMC 2026 Aug 10. (PMC13455339; doi:10.1016/j.canlet.2026.218431)

Supplementary Figure 1

A.

Coeff of Variation	Nuclear area	Cell area
MDA-Parent	90%	278%
MDA-PGCC	134%	149%
HEY-parent	32.3%	50.4%
HEY-PGCC	85.9%	144%

B.

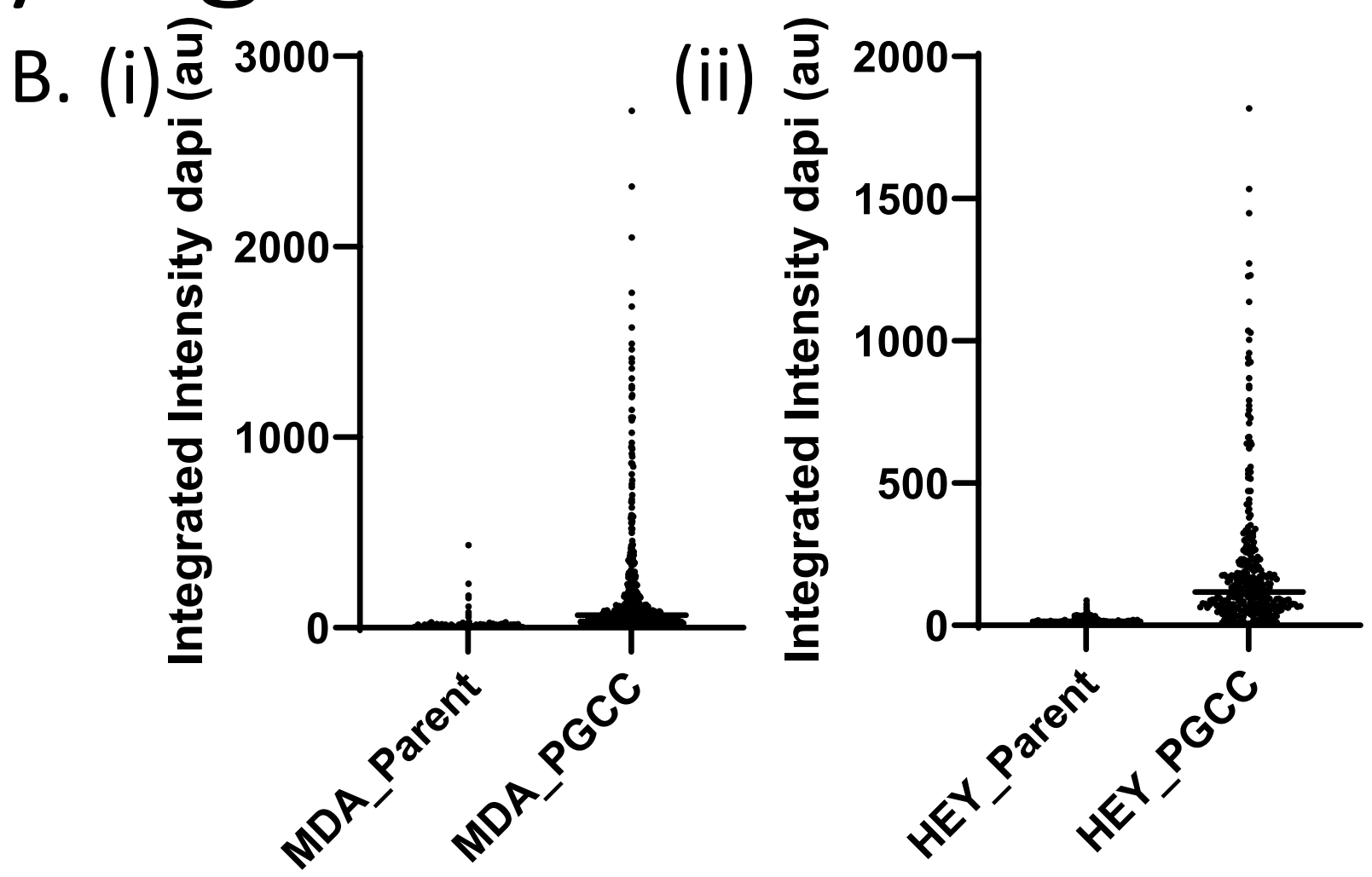

C.

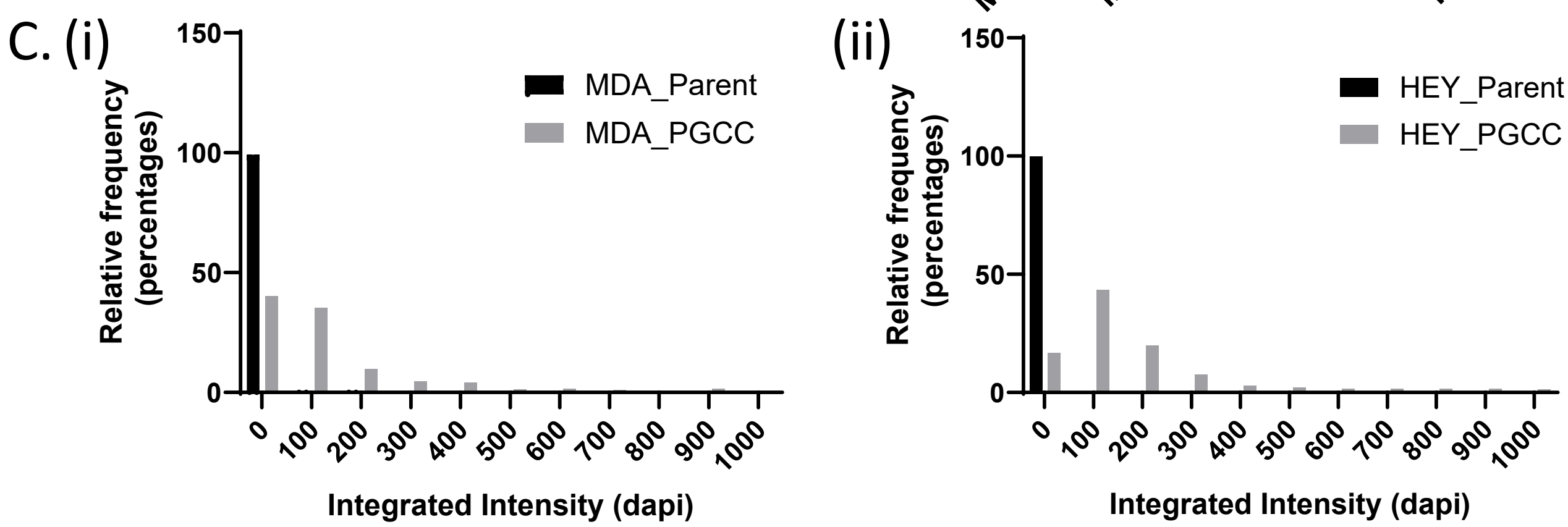

D.

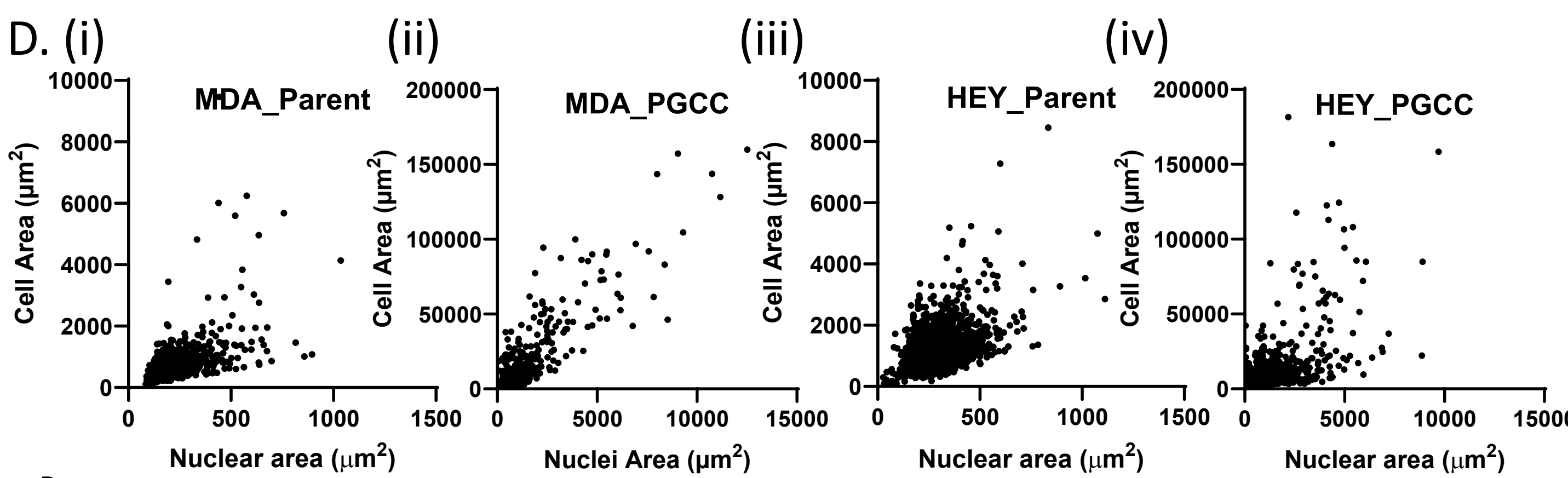

Pearson coefficient(r)	(i)	(ii)	(iii)	(iv)
	0.8866	0.8925	0.3702	0.5386

E.

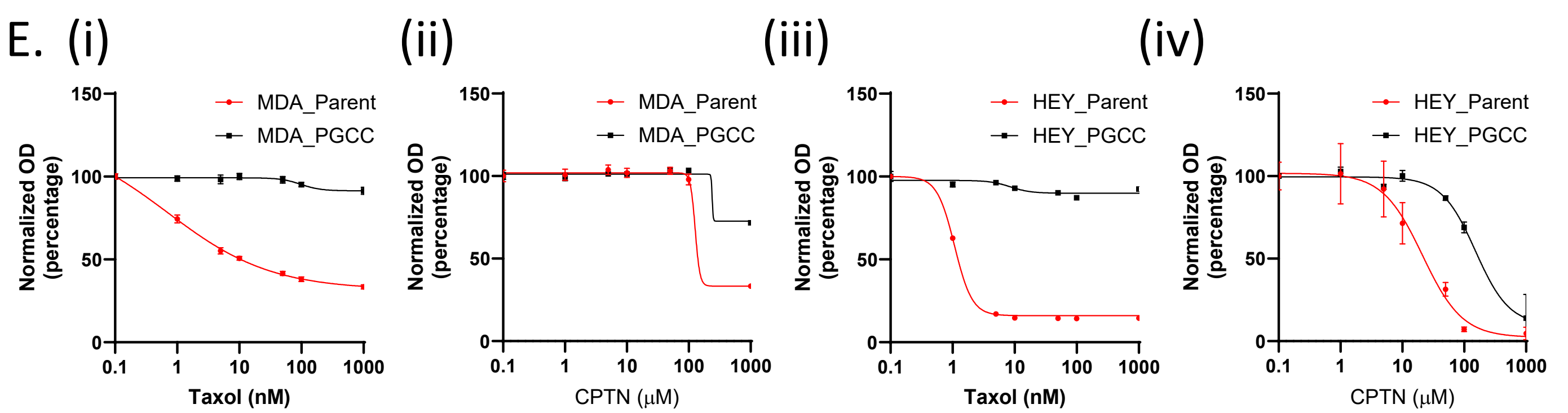

Supplement: Supplementary file 9 [file NIHMS2180034-supplement-9.pdf]
