# Supplementary material for: Autophagy is essential for survival and function of polyploid giant cancer cells under therapeutic stress
Source: Cancer Lett. Author manuscript; Available in PMC 2026 Aug 10. (PMC13455339; doi:10.1016/j.canlet.2026.218431)
Supplement: Supplementary file 10 [file NIHMS2180034-supplement-1.docx]

**Supplementary Figure 1:** Analysis of biophysical properties, nuclear content and chemoresistance in PGCCs. (A) Coefficient of variation in cell and nuclear areas for both parent and paclitaxel (PTX)-induced PGCCs, analyzed across the MDA-MB-231 and HEY cell lines. (B) The total cellular DNA content was quantified by measuring integrated DAPI intensity. Integrated DAPI intensity comparison between parent and PGCCs in MDA-MB-231 (i) and HEY (ii) cell lines. (C) Frequency distribution analysis was employed to compare DNA content between PGCCs and parent cells (i—MDA-MB-231, ii—HEY). This approach enabled the identification of elevated DNA levels in PGCCs relative to their parent cell lines (N=2). (D) Graphs depicting the relationship between cell area and nuclear area presented for parent cells and PGCCs, with MDA-MB-231 data displayed on the left (i-ii) and HEY cell data on the right (iii-iv). Pearson correlation coefficient (r) is indicated below each graph. (E) MTT assay of parent cells and PGCCs after 72 hours of Paclitaxel (PTX) and carboplatin (CPTN) treatment. The error bars represent S.E.M.

**Supplementary Figure 2:** PGCCs in TNBC and HGSOC patient tissue array samples. (A) Sample H&E-stained images of normal ovarian tissue and high grade serous ovarian cancer (HGSOC), with their nuclear boundaries segmented by the Cell Pose-SAM model. (B-D) Tissue microarray analysis was performed on triple negative breast cancer (TNBC) and HGSOC samples to evaluate the nuclear area in both benign and malignant tissues. The analysis also included assessment of the nuclear area of PGCCs, defined as those with nuclear areas exceeding three times the overall mean nuclear area, as well as quantification of PGCCs stratified by disease stage. (D) Association between the proportion of Ki67-positive cells and the quantity of PGCCs in TNBC.

**Supplementary Figure 3:** Molecular phenotype and cell cycle analysis of MDA-MB-231 PGCCs evaluated using Affymetrix microarray (N=4). (A) Principal component analysis (PCA) of genome-wide expression data from Affymetrix arrays comparing MDA-MB-231 parent cells and PGCCs. (B) Unsupervised hierarchical clustering of genes differentially expressed between parent cells and PGCCs. The values shown in color bar are derived from Transcriptome Analysis Console (TAC) output and are reported as “Signal,” which represents normalized probe intensity values following array processing. These values are unitless normalized expression signals rather than raw fluorescence measurements. (C) A list of genes associated with the cell cycle that show reduced expression in PGCCs.

**Supplementary Figure 4:** Pathway analysis of differentially expressed genes in the microarray analysis. (A) PANTHER-GO cellular component classification of significantly regulated genes. (B) Pathways with significant downregulation (z-score < -2) were identified using Ingenuity Pathway Analysis (IPA). (C) *SOD2* gene expression analysis using qRT-PCR in PGCCs relative to parent MDA-MB-231 and HEY cell lines.

**Supplementary Figure 5:** Intracellular distribution analysis of P62 and vimentin in parent and PGCCs. (A) A representative immunofluorescent image shows P62 (magenta) and vimentin (green) distribution in MDA-MB-231-PGCC (Scale bar =20μm). (B) The distribution of intracellular intensity was assessed by segmenting each cell into concentric radial bins, ranging from the nucleus (bin 1) to the cell periphery (bin 8). For each bin, the fractional intensity was determined and subsequently normalized to the corresponding fractional pixel area. Heatmap shows distribution of P62 in MDA-MB-231 PGCCs. (B) Radial intensity distribution of P62 and vimentin in parent cells and PGCCs after 24-hour treatment with vehicle control and 100nM Bafilomycin A1 (BafA1) for both MDA-MB-231 (I, iii) and HEY (ii, iv) cells. (C) Intensity distribution in semi peripheral and peripheral regions of cells was analyzed using bin 5 and bin 8, respectively. Bar graphs display mean ± S.E.M. for n>300 cells per group. Group differences were analyzed using one-way ANOVA with Tukey’s post-hoc test. Statistical significance is indicated as (*) for p<0.05, (***) for p<0.001, and (****) for p<0.0001.

**Supplementary Figure 6:** Effect of chronic autophagy inhibition with BafA1 on PGCCs. (A) The number of nuclei in MDA-MB-231 and HEY PGCCs decreased following autophagy inhibition. (B) PGCCs showed decreased cell and nuclear area variation after prolonged BafA1 treatment. (C) The comparison of cell and nuclear areas in PGCCs following treatment indicates a decrease in the prevalence of large PGCCs. Bar graphs show mean ± S.D. for N=3, n>450 cells per group (unpaired t-test with Welch’s correction, *** p < 0.001, **** p < 0.0001).

**Supplementary Figure 7:** Characterization of daughter cells (DCs) isolated from MDA-MB-231 PGCCs after recovery (N=1). (A) Representative images of DC1 and DC2, stained for nuclei (DAPI), cytokeratin, filamentous-actin and vimentin. Scale bars = 50μm. (B) MTT viability assay in DC1, DC2 and parent cell (PC) populations with varying concentrations of PTX. IC50 values and 95% CI were calculated and indicated in the table below. (C-D) Immunofluorescence image analysis of parent and DC nuclear area (C), mean vimentin (D-i) and cytokeratin intensity (D-ii). Bar graphs show mean ± S.E.M.

**Supplementary Figure 8:** Representative PGCC images showing actin and vimentin structure around p62-tagged micronuclei for MDA-MB-231 (MDA) and HEY cells, respectively. Left panels: scale bar=20um; middle and right panels: scale bars=5um.

**Supplementary Figure 9:** Representative immunofluorescence images and corresponding line-scan analysis (yellow line) illustrating nuclei (cyan) - P62 (magenta) (A) and vimentin (green) (B) intensity distribution in HEY cells. Images were acquired with an Olympus Yokogawa spinning disk confocal microscope with 60x-magnification (Scale bars=20um).

**Supplementary Video 1:** Multinucleated PTX induced MDA-MB-231 PGCCs tracked for 6hours

**Supplementary Video 2:** Directional migration of a PGCC towards scratch wound area (duration: 12 hours)

**Supplementary Video 3:** Migration of daughter cell PGCCs in presence of DMSO (duration: 12 hours)

**Supplementary Video 4:** Migration of daughter cell PGCCs in presence of Bafilomycin A1 (duration: 12 hours)
